# Comparative Genomics of Field Isolates of *Mycobacterium bovis* and *M*. *caprae* Provides Evidence for Possible Correlates with Bacterial Viability and Virulence

**DOI:** 10.1371/journal.pntd.0004232

**Published:** 2015-11-19

**Authors:** José de la Fuente, Iratxe Díez-Delgado, Marinela Contreras, Joaquín Vicente, Alejandro Cabezas-Cruz, Raquel Tobes, Marina Manrique, Vladimir López, Beatriz Romero, Javier Bezos, Lucas Dominguez, Iker A. Sevilla, Joseba M. Garrido, Ramón Juste, Guillermo Madico, Edward Jones-López, Christian Gortazar

**Affiliations:** 1 SaBio, Instituto de Investigación en Recursos Cinegéticos IREC-CSIC-UCLM-JCCM, Ciudad Real, Spain; 2 Department of Veterinary Pathobiology, Center for Veterinary Health Sciences, Oklahoma State University, Stillwater, Oklahoma, United States of America; 3 Departamento de Sanidad Animal, Facultad de Veterinaria, Universidad Complutense de Madrid, Madrid, Spain; 4 Center for Infection and Immunity of Lille (CIIL), INSERM U1019 –CNRS UMR 8204, Université de Lille, Institut Pasteur de Lille, Lille, France; 5 Oh no sequences! Research Group, Era7 Bioinformatics, Granada, Spain; 6 Centro de Vigilancia Sanitaria Veterinaria (VISAVET), Universidad Complutense de Madrid, Madrid, Spain; 7 MAEVA SERVET S.L., Madrid, Spain; 8 NEIKER-Tecnalia, Instituto Vasco de Investigación y Desarrollo Agrario, Departamento de Sanidad Animal, Vizcaya, Spain; 9 Section of Infectious Diseases, Department of Medicine, Boston Medical Center and Boston University School of Medicine, Boston, Massachusetts, United States of America; University of California San Diego School of Medicine, UNITED STATES

## Abstract

Mycobacteria of the *Mycobacterium tuberculosis* complex (MTBC) greatly affect humans and animals worldwide. The life cycle of mycobacteria is complex and the mechanisms resulting in pathogen infection and survival in host cells are not fully understood. Recently, comparative genomics analyses have provided new insights into the evolution and adaptation of the MTBC to survive inside the host. However, most of this information has been obtained using *M*. *tuberculosis* but not other members of the MTBC such as *M*. *bovis* and *M*. *caprae*. In this study, the genome of three *M*. *bovis* (MB1, MB3, MB4) and one *M*. *caprae* (MB2) field isolates with different lesion score, prevalence and host distribution phenotypes were sequenced. Genome sequence information was used for whole-genome and protein-targeted comparative genomics analysis with the aim of finding correlates with phenotypic variation with potential implications for tuberculosis (TB) disease risk assessment and control. At the whole-genome level the results of the first comparative genomics study of field isolates of *M*. *bovis* including *M*. *caprae* showed that as previously reported for *M*. *tuberculosis*, sequential chromosomal nucleotide substitutions were the main driver of the *M*. *bovis* genome evolution. The phylogenetic analysis provided a strong support for the *M*. *bovis/M*. *caprae* clade, but supported *M*. *caprae* as a separate species. The comparison of the MB1 and MB4 isolates revealed differences in genome sequence, including gene families that are important for bacterial infection and transmission, thus highlighting differences with functional implications between isolates otherwise classified with the same spoligotype. Strategic protein-targeted analysis using the ESX or type VII secretion system, proteins linking stress response with lipid metabolism, host T cell epitopes of mycobacteria, antigens and peptidoglycan assembly protein identified new genetic markers and candidate vaccine antigens that warrant further study to develop tools to evaluate risks for TB disease caused by *M*. *bovis/M*.*caprae* and for TB control in humans and animals.

## Author Summary

Mycobacteria belonging to the *Mycobacterium tuberculosis* complex infect humans and animals since pre-history and are a serious health problem worldwide. Whole-genome sequencing and comparative genomics generate information on the evolution and molecular basis of pathogenicity and transmissibility. However, while genomic information is increasingly available for the main human pathogens such as *Mycobacterium tuberculosis*, little is known about closely related bacteria, *Mycobacterium bovis* and *Mycobacterium caprae*. These mycobacteria infect humans causing zoonotic tuberculosis and are the main causative agents of animal tuberculosis. Although human-to-human transmission of zoonotic tuberculosis is limited, the infection often causes extra-pulmonary disease in humans and is still a major public health concern in developing countries, causing not only human disease but also severe effects on livelihoods. In this study, whole-genome sequences and targeted comparative genomics of three *Mycobacterium bovis* and one *Mycobacterium caprae* field isolates generated new information on the evolution and phenotypic variation of these mycobacteria. The results identified new genetic markers and candidate vaccine antigens that warrant further study to develop tools to evaluate risks for tuberculosis caused by *M*. *bovis/M*.*caprae* and for disease control in humans and animals.

## Introduction

*Mycobacterium tuberculosis* has infected more than 2.5 billion people worldwide with approximately 9 million new tuberculosis (TB) cases reported every year [1]. Animal TB is caused by infection with *Mycobacterium bovis* and closely related members of the *M*. *tuberculosis* complex (MTBC) such as *M*. *caprae*. Although cattle are the main concern regarding animal TB in industrialized countries, several other mammals including humans are also infected [2,3]. Eurasian wild boar (*Sus scrofa*) are a natural reservoir for *M*. *bovis* in some regions and thus vaccination strategies are being developed for TB control in this species [4–8]. Several other domestic and wild animals are also infected with *M*. *bovis* and may act as reservoir species [9–13].

The life cycle of mycobacteria is complex and the mechanisms resulting in pathogen infection and survival in host cells are not fully understood [14]. Nevertheless, it is generally accepted that after inhalation into the lung or entry to the oropharyngeal cavity, the principal entry routes, mycobacteria of the MTBC are phagocytized by macrophages, which constitute the main host cell. As with other intracellular bacteria, mycobacteria survive inside macrophages by escaping host immune response, which results in the formation of a granuloma that effectively contains infected cells. A change in the host-bacterial equilibrium of granulomas is thought to result in the release of infected cells outside containment and onward transmission of mycobacteria to susceptible hosts [14].

The association between *M*. *bovis* spoligotypes and TB lesions in cattle has been used to correlate bacterial genotype with virulence [15]. However, these genotyping methods cover only a small portion of the approximately 4,000 genes contained in the 4.4 Mb mycobacterial genome [16]. Recently, whole-genome sequencing and comparative genomics analyses have provided new insights into the evolution and adaptation of the MBTC to survive inside the host and explained phenotypic traits related with transmissibility and virulence [16–21]. Although the first *M*. *tuberculosis* genome sequence was reported in 1998 [16], it was not until 2003 when the first *M*. *bovis* genome was sequenced [19]. Presently, a large number of *M*. *tuberculosis* but few *M*. *bovis* (except for BCG strains) genome sequences are available [18]. The relative paucity of *M*. *bovis* genome sequence information limits the possibility of characterizing mycobacterial evolution and correlation with virulence at the whole-genome level.

In this study, the genome of three *M*. *bovis* (MB1, MB3, MB4) and one *M*. *caprae* (MB2) field isolates with different lesion score, prevalence and host distribution phenotypes were sequenced. Genome sequence information was used for whole-genome and protein-targeted comparative genomics analysis with the aim of finding correlates of phenotypic variation with potential implications for TB disease risk assessment and control.

## Materials and Methods

### Ethics statement

All animal sampling was post-mortem. Wildlife samples came from hunter-harvested individuals that were shot during the legal hunting season independently and prior to our research while livestock samples were obtained at the slaughterhouse where they were being processed as part of the normal work and submitted to the reference laboratory by the slaughterhouse veterinarian. According to EU and National legislation (2010/63/UE Directive and Spanish Royal Decree 53/2013) and to the University of Castilla–La Mancha guidelines no permission or consent is required for conducting this type of study. Field isolates used come from the EU Reference Laboratory for Animal Tuberculosis (VISAVET).

### Mycobacteria isolates

Three *M*. *bovis* (MB1, MB3, MB4) and one *M*. *caprae* (MB2) field isolates were selected for this study (Table 1). These isolates were originally obtained from wild boar (MB3, MB4), cattle (MB1) and goat (MB2). The study focused on Ciudad Real Province, Spain. This is a high ungulate density area, the west side of the province composed by interspersed game ranges and protected nature areas, with persistent TB infection in extensive livestock farms [22]. Nine hundred MTBC isolates collected from wild ungulates and livestock from 2000 to 2011 were spoligotyped resulting in 62 different spoligotypes ([23] and S1 Fig).

**Table 1:** Mycobacteria isolates used in the study.

Species, spoligotype (ID)	Distribution (isolation frequency)	Hosts	TB lesion score
*M*. *bovis*, SB0339 (MB1)	High	C, G, D, F, W, P	Low
*M*. *caprae*, SB0157 (MB2)	Medium	C, G, S, D, W, P	High
*M*. *bovis*, SB0134 (MB3)	High	C, G, D, F, W, P, S, B	High
*M*. *bovis*, SB0339 (MB4)	High	C, G, D, F, W, P	Low

Summary of the results obtained after the analysis of different MTBC spoligotypes fulfilling selection criteria (i-iii) described in Materials and Methods. The spoligotypes SB0134, SB0339 and SB0157 were among the most frequent spoligotypes found in both livestock and wild ungulates in the study area. The spoligotypes SB0134 and SB0339 clustered in space and time and showed high abundance. Findings in Iberian red deer showed a higher lesion score caused by spoligotype SB0134 when compared to spoligotype SB0339 while these spoligotypes and particularly SB0134 and SB0157 suggested an association with high TB severity in Eurasian wild boar.

Abbreviations: C, cattle; G, goat; D, deer; F, fallow deer; W, wild boar; P, pig; S, sheep; B, badger.

The criteria for selection of the MTBC spoligotypes included in the study were based on:

- Inclusion into an emergent spoligotype cluster defined considering the estimated evolutionary history of the strains belonging to it. Spacer oligonucleotide typing, or spoligotyping, is a genotyping method used to study the epidemiology of the MTBC and it is based on the presence or absence of 43 spacers at the direct repeat locus [24]. The visualization of spoligotype patterns based on an estimated evolutionary history is a procedure to predict emerging strains or genotypes associated with elevated transmission and can be appropriate for medium-large spatio-temporal scale analysis. This method was used to determine if *M*. *tuberculosis* strains were spreading faster than the background transmission rate [25] and implemented in DESTUS (Detecting Emerging Strains of Tuberculosis Using Spoligotypes), which is included in spolTools (http://www.emi.unsw.edu.au/spoltools/). This method was designed to be used with self-contained datasets corresponding to specific regions, rather than composite data from different countries or collection periods. We conducted these analyses for the total and for each host species separately.
- Inclusion into a spoligotype cluster that successfully clusters in space and time. We combined the approach (i) with cluster analyses (purely spatial and spatio-temporal as implemented in SatScan software version 5.1.3; [26]) at the population (wildlife management area or livestock farm) level to evidence emerging strains to select spoligotypes that successfully clustered in space and time. The Bernoulli purely spatial and spatio-temporal models were used to detect spoligotype clusters in individual farms or wildlife management areas. Spoligotypes from non-clustered strains were used as controls.
- For those spoligotypes fulfilling criteria (i) and (ii), a third criterion based on TB severity in natural infections was considered. Firstly, TB severity in wild boar, the key reservoir host for *M*. *bovis* and *M*. *caprae* in Iberia [5, 27] was used based on the distribution of spoligotypes SB0134, SB0157, and SB0339 by age-class in naturally infected wild boar from Montes de Toledo, Spain. Secondly, we also considered the relationship between TB lesion scores and spoligotypes in red deer (*Cervus elaphus*), also a reservoir species for *M*. *bovis* in Iberia [28].

Following these three criteria, the *M*. *bovis* spoligotypes SB0339 (MB1, MB4) and SB0134 (MB3) were selected. The *M*. *caprae* spoligotype SB0157 (MB2) was included in the study as an outgroup but closely related species [29] and for the increased proportion of *M*. *caprae* isolated from bovine samples during 2004–2009 [30]. The MB4 isolate was included because although it has the same spoligotype as MB1, it served as a model to characterize possible differences between MTBC isolates otherwise classified with the same spoligotype.

### Bacterial growth and genomic DNA extraction

The four isolates were grown in 15 ml of Middlebrook 7H9 liquid media supplemented with 0.36% sodium pyruvate and 10% OADC (Oleic Albumin Dextrose Catalase) for 5 weeks. Chromosomal DNA samples were obtained as described by van Soolingen et al. [31]. Briefly, cultures were centrifuged and pellets were washed twice in 5 ml water. Mycobacteria were heated at 100°C for 15 min to kill the cells. After centrifugation, the cells were resuspended in 5 ml TE buffer (0.01 M Tris-HCl, 0.001 M EDTA, pH 8.0). Lysozyme was added to a final concentration of 1 mg/ml and the tube was incubated over night at 37°C. Eighty hundred and seventy-five microliters of 10% sodium dodecyl sulfate (SDS) with 62.5 μl of proteinase K (at a 10-mg/ml concentration) were added, and the mixture was incubated for 1 h at 60°C. The extract was transferred to a phase lock gel tube (prime5, Fisher Scientific SL, Madrid, Spain) for a phenol/chloroform DNA extraction.

### Genome sequencing

Genomic DNA (2–5 μg) was subjected to mechanical fragmentation using a BioRuptor (Life Technologies, Carlsbad, CA, USA). The number of cycles was adjusted to obtain DNA fragments of a final average size of about 500 pb. Samples were then used to prepare sequencing-amenable TruSeq libraries (NEB-Next, New England Biolabs, Ipswich, MA, USA). Briefly, DNA fragments were made blunt-ended, phosphorylated, adenylated and Illumina-compatible adapters were ligated. After purification, barcoded sequences as well as Illumina-specific sequences were introduced by PCR, followed by quantitation of individual libraries. Libraries were then pooled and quantified again. A quality control of the pooled library made in bioanalyzer is shown in the figure, including an estimation of the percentage of non-overlapping reads that could be obtained using a 2x250 paired-end sequencing protocol. Library was qPCR-quantitated and brought to a final concentration of 10 nM. DNA was then denatured and equilibrated so that a final concentration of 18 pM of library was loaded onto a MiSeq v.3 flowcell (Illumina, San Diego, CA, USA) and sequenced using a 2x250 paired-end sequencing protocol to obtain more than 400x high quality coverage (1.9–2.5 Gb) with 84% of the bases showing a Q30 factor > 30. Reads were finally split according to barcodes and used for bioinformatics analysis.

### Genome sequence assembly and annotation

High quality overlapping reads were merged using FLASH (Magoc et al., 2011) and then assembled using Velvet [32] with k-value = 97 (S1 Table). Contigs were annotated using BG7 [33,34] (S2 Table). For annotation, a set of 191,017 reference proteins was used including (a) all Uniprot proteins from *M*. *bovis* and *M*. *tuberculosis*, (b) a set of bacterial antibiotic resistance related Uniprot proteins selected using the GO annotation terms “antibiotic resistance” and “response to antibiotic” and a selection of proteins based on similarity to the proteins of ARDB [35], and (c) all Uniprot proteins with Enzyme Code (EC) from MTBC. For whole genome comparative analysis, the 4 genomes were then aligned against the *M*. *bovis* reference genome sequence (AF2122/97; http://www.ncbi.nlm.nih.gov/nuccore/31791177) using Differences program (Turrientes et al., 2010) that allows comparisons at the whole genome level and particularly the detection of substitutions, insertions or deletions of any length and at any region of the genome. Genome sequence information and annotation was deposited in GenBank under the accession numbers CDHF01000001-CDHF01000049, CDHG01000001-CDHG01000059, CDHH01000001-CDHH01000094 and CDHE01000001-CDHE01000118 for MB1-MB4 isolates, respectively [36].

### Whole genome phylogenetic analysis

A phylogenetic tree was constructed based on the SNPs found at the core genome sequence shared with a similarity over a threshold between all genomes included in the analysis using Harvest [37] and visualized with EvolView (v 198.3) [38]. Harvest defines the SNPs aligning whole assembled genomes and not reads like in other approaches, thus allowing the identification of the gene or the intergenic region where differences are allocated. The SNPs are included in the.vcf file provided by Harvest suite. The genomes used in the SNP phylogenetic analysis using Harvest included MB1-MB4, *M*. *bovis* AF2122/97 (http://www.ncbi.nlm.nih.gov/nuccore/31791177), *M*. *bovis* ATCC BAA-935 (http://www.ncbi.nlm.nih.gov/nuccore/690294709), *M*. *bovis* BCG Pasteur 1173P2 (http://www.ncbi.nlm.nih.gov/nuccore/121635883), and *M*. *tuberculosis* H37Rv (http://www.ncbi.nlm.nih.gov/nuccore/448814763).

### Multilocus SNP phylogenetic analysis

The multilocus sequence analysis was conducted using 18 genes coding for the proteins linking stress response with lipid metabolism (S3 Table). The nucleotide sequences of the genes were obtained from the genomes of MB1-MB4 isolates used in this study. For comparison, the same sequences were obtained from the reference *M*. *bovis* BCG Pasteur 1173P2, *M*. *bovis* AF2122/97 and *M*. *tuberculosis* H37Rv. *M*. *canettii* (NCBI reference sequence NC_019950) was used as outgroup. The nucleotide sequences were concatenated and then aligned with MAFFT (v7), configured for the highest accuracy [39]. After alignment, regions with gaps were removed and 20080 gap-free sites were used in maximum likelihood phylogenetic analysis as implemented in PhyML (v3.0 aLRT) [40,41]. The reliability for the internal branches was assessed using the approximate likelihood ratio test (aLRT–SH-Like) [41]. Graphical representation and editing of the phylogenetic trees were performed with EvolView (v 198.3) [38]. The alignments obtained by MAFFT were used to perform codon alignments using HIV database website (www.hiv.lanl.gov; [42]). Non-synonymous (dN) and synonymous (dS) nucleotide substitutions were classified using the SNAP method [43] implemented in HIV database website [42]. SNPs were identified by pairwise comparison of MB1-MB4 and *M*. *bovis* AF2122/97 using SNAP.

### Nucleotide substitution rate analysis

The dN/dS ratio was calculated for the 18 genes coding for the proteins linking stress response with lipid metabolism (S3 Table) and for 81 antigen-coding genes (S4 Table) present on each of the MB1-MB4 isolates included in the study. Under the Datamonkey server (http://www.datamonkey.org; [44]), the algorithm SLAC [45] was used to detect which nucleotide substitution site were positively or negatively selected. For each dS and non-synonymous dN substitution site, four measurements were made: normalized expected (ES and EN) and observed numbers (NS and NN). The SLAC algorithm then calculated: dN = NN/EN and dS = NS/ES. If dN < dS a codon was negatively selected and if dN > dS a codon was positively selected. A two-tailed extended binomial distribution set at P<0.05 was used to assess significance of the algorithm. The SLAC algorithm uses a neighbor-joining tree with a maximum likelihood for branch lengths and substitution rates.

### Sequence analysis of Rv0050

To identify non-synonymous mutations that may be associated with virulence and/or transmission, the orthologs of peptidoglycan assembly protein locus Rv0050 (H37Rv) in strains MB1-MB4 were compared to the equivalent locus in animal isolates (BCG Pasteur 1173P2, AF2122/97, 09–1192) and human isolates (Bz 3115, B2 7505) of *M*. *bovis* available in the GenBank. The *M*. *bovis* strains were selected among all strains for having distinct Rv0050 locus and/or distinct source of isolation. The presence of a putative signal peptide and cleavage site in the Rv0050 locus was analyzed with a previously validated program for their prediction in *M*. *tuberculosis* (SignalP; http://www.cbs.dtu.dk/services/SignalP-3.0/) [46]. Results were also confirmed with two other programs, Signal-Blast (http://sigpep.services.came.sbg.ac.at/signalblast.html) and Phobious (http://phobius.sbc.su.se/).

### PCR and sequence analysis

To confirm selected SNPs identified in the mycobacteria genomes sequenced in this study, sequence-specific oligonucleotide primers were design using reference genomes *M*. *bovis* AF2122/97 (BX248333.1) or *M*. *tuberculosis* H37Rv (AL123456.3) for PCR and sequencing of the amplicons. Selected loci and direct and reverse primers used for analysis included Rv0050 (ponA1; 5´-GACTTTCCCCAAACCGACCGAGG-3’ and 5’-GATCGGTCCCCCGACCACCATT-3’), Rv0589 (MCE2a; 5´-GTGCCAACGCTGGTGACGAG-3’ and 5’-AGAACACGATCAACCCATGA-3’), Rv1198 (ESAT-6; 5´-ATGACCATCAACTATCAATT-3’ and 5’-TCGGCTCCAGCTGGGCCTGA-3’), and Rv1860 (FAP-B; 5´-ATGCATCAGGTGGACCCCAA-3’ and 5’- AGCGGACCTTACCGGCCTGA-3’). The PCR was conducted using 2 μl of DNA with 20 pmol of each primer in a 50 μl reaction PCR Master Mix (Promega, Madison, WI, USA) using a GeneAmp PCR System 2700 thermocycler (Applied Biosystems, Carlsbad, CA, USA). PCR products were electrophoresed on 1.5% agarose gels to check the size of the amplified fragments by comparison to molecular weight marker GeneRuler 1kb DNA ladder (Thermo Scientific, Waltham, MA, USA). Amplified DNA fragments were purified with a PureLink Quick PCR Purification Kit (Thermo Scientific, Waltham, MA, USA) and sequenced using the reverse primer on each locus. Amplicons from at least two independent PCR reactions were sequenced.

## Results and Discussion

### Selection of mycobacteria isolates

Among the 900 MTBC field isolates analyzed, 62 different spoligotypes were identified suggesting that the study area is one of the regions with the highest diversity of MTBC spoligotypes described in the literature [47]. The high genetic diversity of MTBC in this area supported an important natural scenario where MTBC and particularly *M*. *bovis* have diversified, thus offering an interesting epidemiological and evolutionary context where new genotypes can emerge and diversify in terms of adaption to host under a range of environmental and human driven factors.

**Fig 1:**
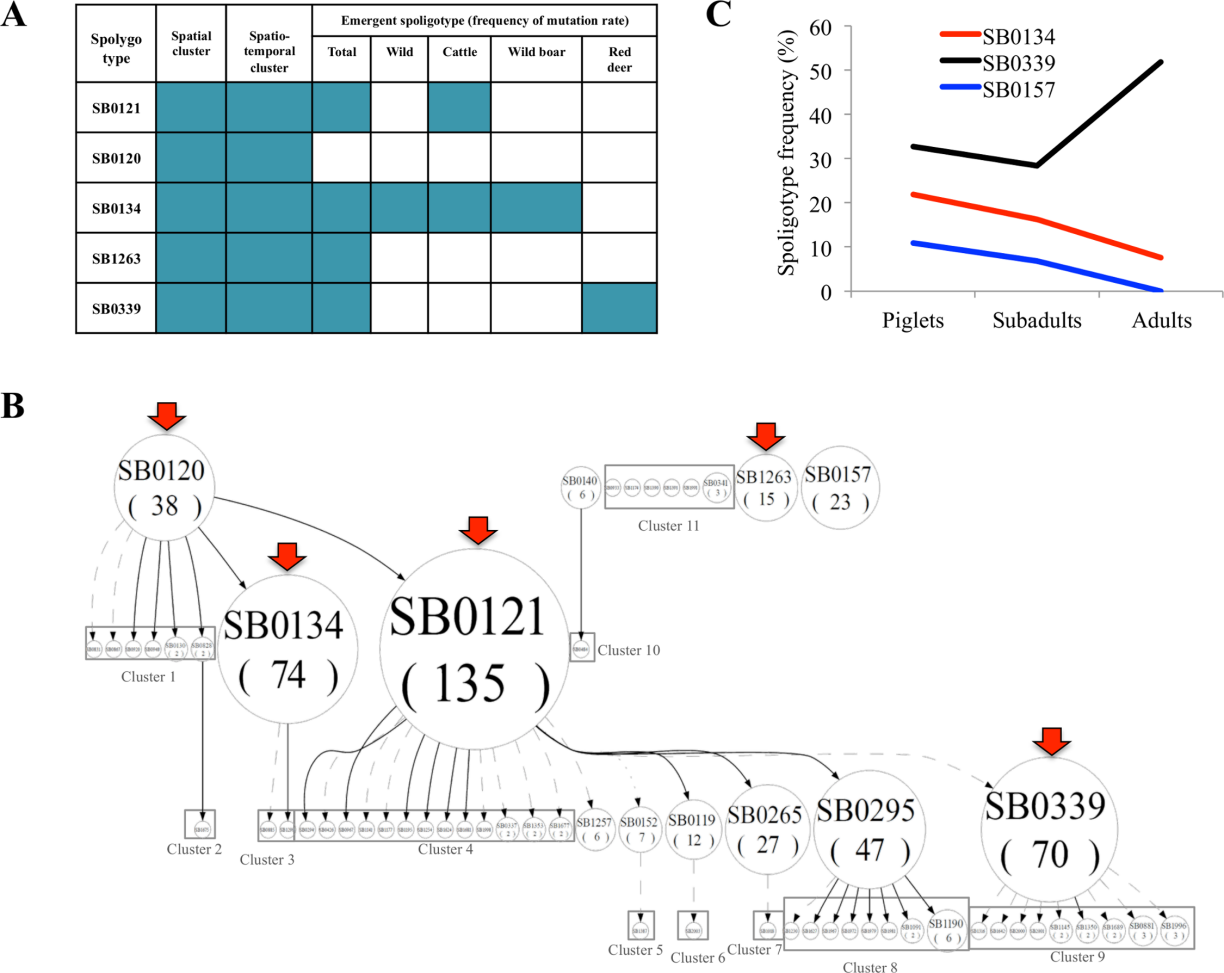
Selection of mycobacteria isolates. (A) Potentially emerging spoligotypes according to different criteria of emergence: (i) spatial and spatio-temporal clustering and (ii) the frequency of mutation rate (abundance for the expected mutation rate) considering all the hosts together and separately. Both criteria are indicative of high transmissibility and emergence. (B) The hierarchical tree showing a history of mutation events and relationship among spoligotypes in a sample of isolates from south central Spain (62 different spoligotypes out of 900 different MTBC isolates from livestock and wildlife). The size of each node is proportional to the number of isolates (the cluster size); edges between nodes reflect evolutionary relationships between spoligotypes with arrowheads pointing to descendants. Spoligotypes that are spreading faster than the background rate are marked with arrows. Spoligotypes that are inferred to be derived from another spoligotype are placed below the inferred parent. Cluster 1: SB0831, SB0867, SB0920, SB0948, SB0130(2), SB0828 (2); Custer 2: SB1675; Cluster 3: SB0885, SB1299; Cluster 4: SB0294, SB0426, SB0967, SB1141, SB1177, SB1195, SB1254, SB1624, SB1681, SB1998, SB0337(2), SB1353(2), SB1677(2); Cluster 5: SB1387; Cluster 6: SB2003; Cluster 7: SB1018; Cluster 8: SB1230, SB1627, SB1967, SB1972, SB1979, SB1981, SB1091 (2), SB1190 (6); Cluster 9: SB1316, SB1642, SB2000, SB2001, SB1145(2), SB1350 (2), SB1689 (2), SB0881 (3), SB1996 (3); Cluster 10: SB0484; Cluster11: SB0933, SB1174, SB1390, SB1391, SB1991, SB0341(3). (C) Distribution of spoligotypes SB0134, SB0157 and SB0339 by age-class in naturally infected Eurasian wild boar from Montes de Toledo, Spain. The percentage of detection of SB0339 in adult wild boar was higher than in yearlings and juveniles, indicating an age-increasing infection. By contrast, the percentage of adults infected with SB0134 was lower than expected, and no SB0157 infected adults were found, indicating that yearling or juvenile wild boar infected with these strains had a low chance of survival.

The spoligotypes SB0121, SB0134, SB0339, SB0120, SB1263 and SB0157 were among the most frequent ones found in both livestock and wild ungulates in the study area (S1 Fig). The spoligotypes found at a higher frequency than expected given a predicted mutation rate were identified (Fig 1A) and represented in a hierarchical tree (Fig 1B). The spoligotypes SB0134 (MB3) and SB0339 (MB1 and MB4), which clustered in space and time and showed low mutation rates but high abundance, were selected as emergent under study conditions. The output hierarchical tree suggested a history of mutation events and a relationship between these spoligotypes with different levels of delection for spoligotypes SB0134 (MB3) and SB0339 (MB1 and MB4) (Fig 1B). These spoligotypes were therefore chosen for this study as fulfilling selection criterion (i) and (ii) described above. Additionally, findings in Iberian red deer showed a higher lesion score caused by spoligotype SB0134 when compared to spoligotype SB0339 (Mann-Whitney U test; p = 0.04) while these spoligotypes and particularly SB0134 and SB0157 suggested an association with high TB severity in Eurasian wild boar thus also fulfilling selection criteria (iii) (Fig 1C).

In summary, three *M*. *bovis* (MB1, MB3, MB4) and one *M*. *caprae* (MB2) field isolates with different prevalence, lesion score and host distribution phenotypes were selected for this study (Table 1). MB3 showed high distribution and lesion score while MB1 and MB4 were highly distributed but with low lesion score. The *M*. *caprae* (MB2) isolate had moderate distribution and high lesion score and was selected for comparison with the *M*. *bovis* isolates. These phenotypic variations are relevant for pathogen transmission and virulence and could be correlated with genome sequence information with implications for TB disease risk assessment and control.

### Phylogenetic analysis and comparative genomics

**Fig 2:**
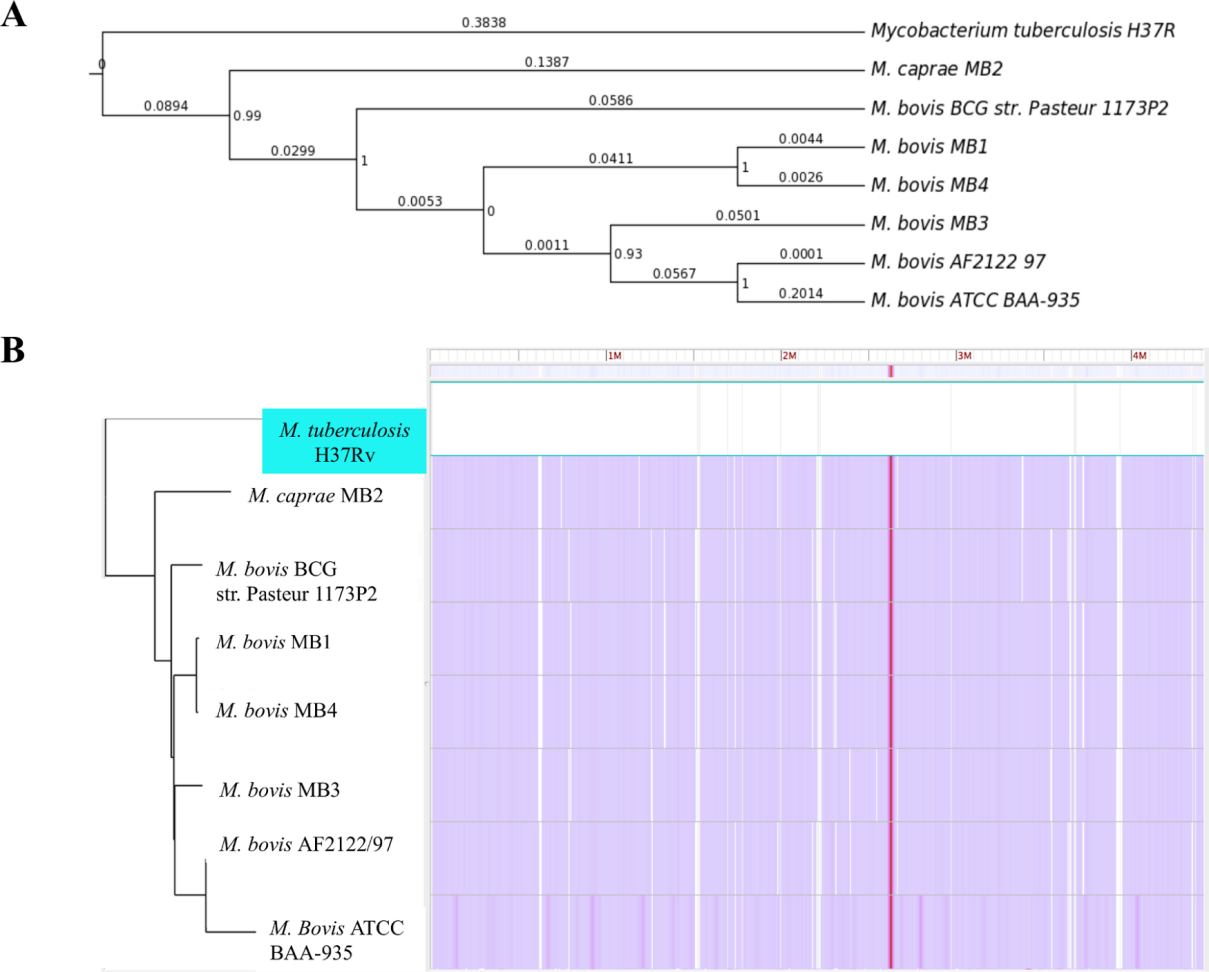
Phylogenetic analysis. (A) The phylogenetic tree showing the bootstrap scores (at the bifurcations) and the branchlength values (over the branches) was based on the SNPs included in the.vcf file provided by Harvest suite as the result of analyzing the core-genome alignment of the 7 genomes included in the phylogenetic analysis (*M*. *tuberculosis* H37Rv was used as reference). The image was built using EvolView. (B) Screenshot of Harvest suite tool displaying the phylogenetic tree and the core-genome multiple alignment for the 7 genomes included in the analysis. Regions with sequence differences and discarded from the SNP analysis are shown in white. The regions included in the SNP analysis are colored with a similarity dependent color scale.

The results of the phylogenetic analysis showed that the MB1 and MB4 isolates with the same spoligotype were the most closely related isolates (Figs 2A and S2). The MB2 isolate (*M*. *caprae*) clustered separately from the other isolates, which clustered together with *M*. *bovis* sequences. Nevertheless, *M*. *caprae* (MB2) was closely related to *M*. *bovis* when compared to *M*. *tuberculosis* (Fig 2A).

**Fig 3:**
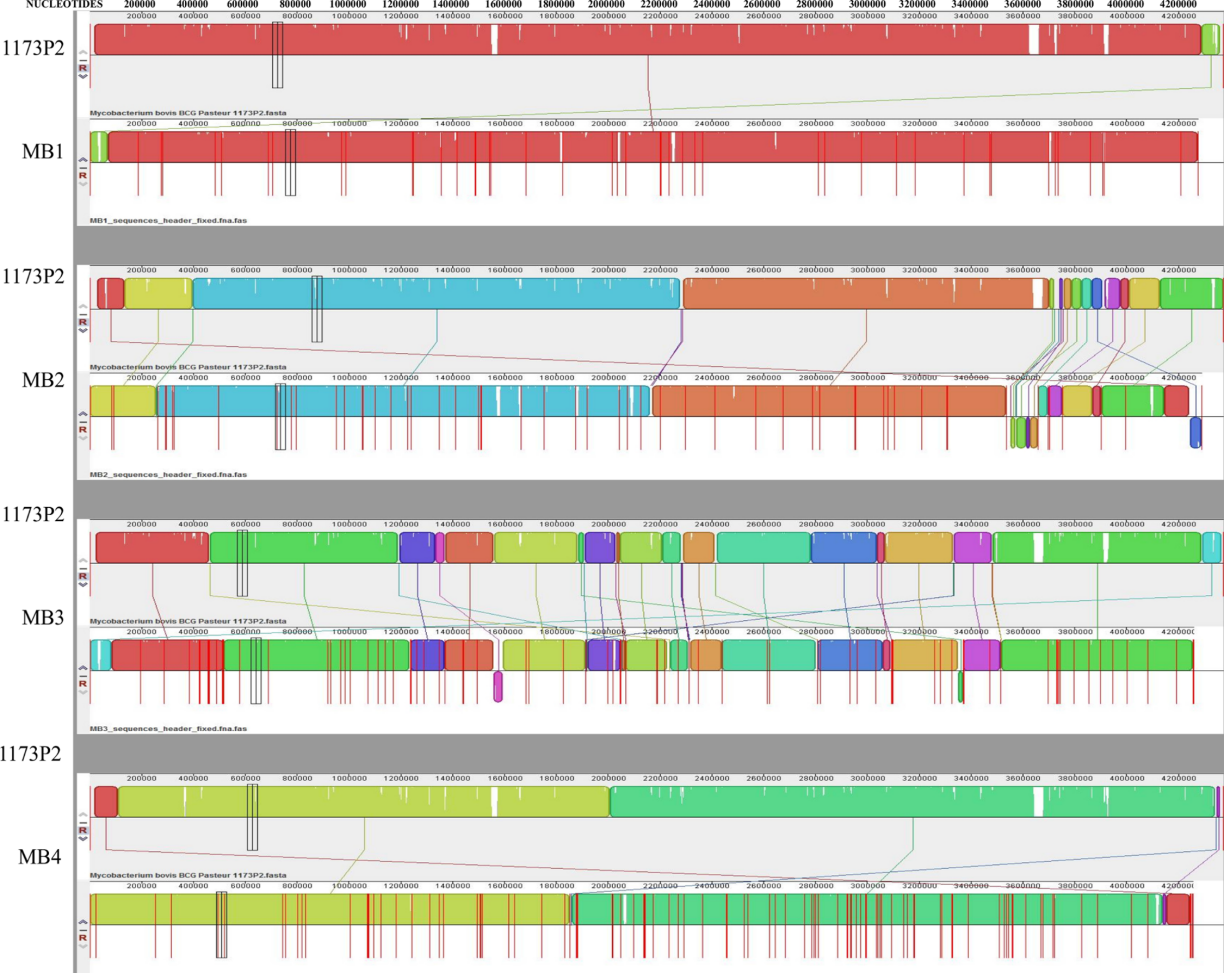
Comparative genomics of MB1-MB4 isolates. Genome sequence comparison between mycobacteria isolates MB1-MB4 and the most similar reported sequence of *M*. *bovis* BCG Pasteur 1173P2 using Differences program.

The genome of *M*. *bovis* BCG Pasteur 1173P2 was the most similar to all sequenced mycobacteria genomes (Figs 2A, 2B and 3). It has been proposed that during evolution, a clone of *M*. *tuberculosis* that was originally adapted to cause human TB evolved to infect a non-human mammal and thus began the transition into non-human ecotypes such as *M*. *bovis*, which in turn spread to cattle, goats, oryx, seals and pigs [20]. Our results supported a close relationship between *M*. *bovis* isolates and suggested that *M*. *caprae* is one of the *M*. *bovis*-related mycobacteria adapted to infect goats and sheep as well as other hosts such as wild boar, red deer, cattle and humans [29,48,49].

To better understand the relationship between these isolates, a comparative genomics approach was used. The results showed the presence of translocations, deletions of small genomic regions and SNPs between genomic sequences (Figs 2B, 3 and S2). Large-scale polymorphism studies have demonstrated that the MTBC shows a large number of deletions of small genomic regions consistent with the reductive evolution typical of intracellular bacteria [20]. Nevertheless, sequential chromosomal nucleotide substitutions are considered to be the main driver in the *M*. *tuberculosis* genome evolution [20]. The results reported here supported these findings for *M*. *bovis* isolates and suggested that the isolates with high lesion score, MB2 and MB3, contained the largest number of polymorphisms when compared to the MB1 and MB4 isolates with low lesion score (Figs 2B and 3). However, a clear correlation between phenotype and genome sequence requires a protein-targeted comparative analysis between the different isolates. For protein-targeted analysis, the study was focused on proteins that are known to play an important role in mycobacterial viability or virulence.

### Protein-targeted comparative analysis

Protein-targeted comparative analysis was conducted for (a) the ESX or type VII secretion system, (b) proteins linking stress response with lipid metabolism, (c) host T cell epitopes of mycobacteria, (d) antigens and (e) peptidoglycan assembly protein to define possible correlates with bacterial virulence and viability or distribution.

#### (a) ESX secretion system family proteins

The ESX or type VII secretion system (ESX-1 through ESX-5) proteins were targeted for analysis due to their role in pathogen-host interactions affecting mycobacterial viability (ESX-3, ESX-5 loci) or virulence (ESX-1, ESX-5 loci) and as candidate protective antigens [50,51]. The ESX locus exists as either complete or partial cluster of ESX-coding genes and other components [50–52]. Each ESX complete locus contains 2 ESX secreted protein *Esx* genes, 4 ESX core component *Ecc* genes and additional ESX secretion-associated protein *Esp* genes [50]. Based on the current model described for the ESX system, Esx heterodimers are recognized by EccC, which provides energy to propel substrates through the transmembrane export channel EccD [50].

**Fig 4:**
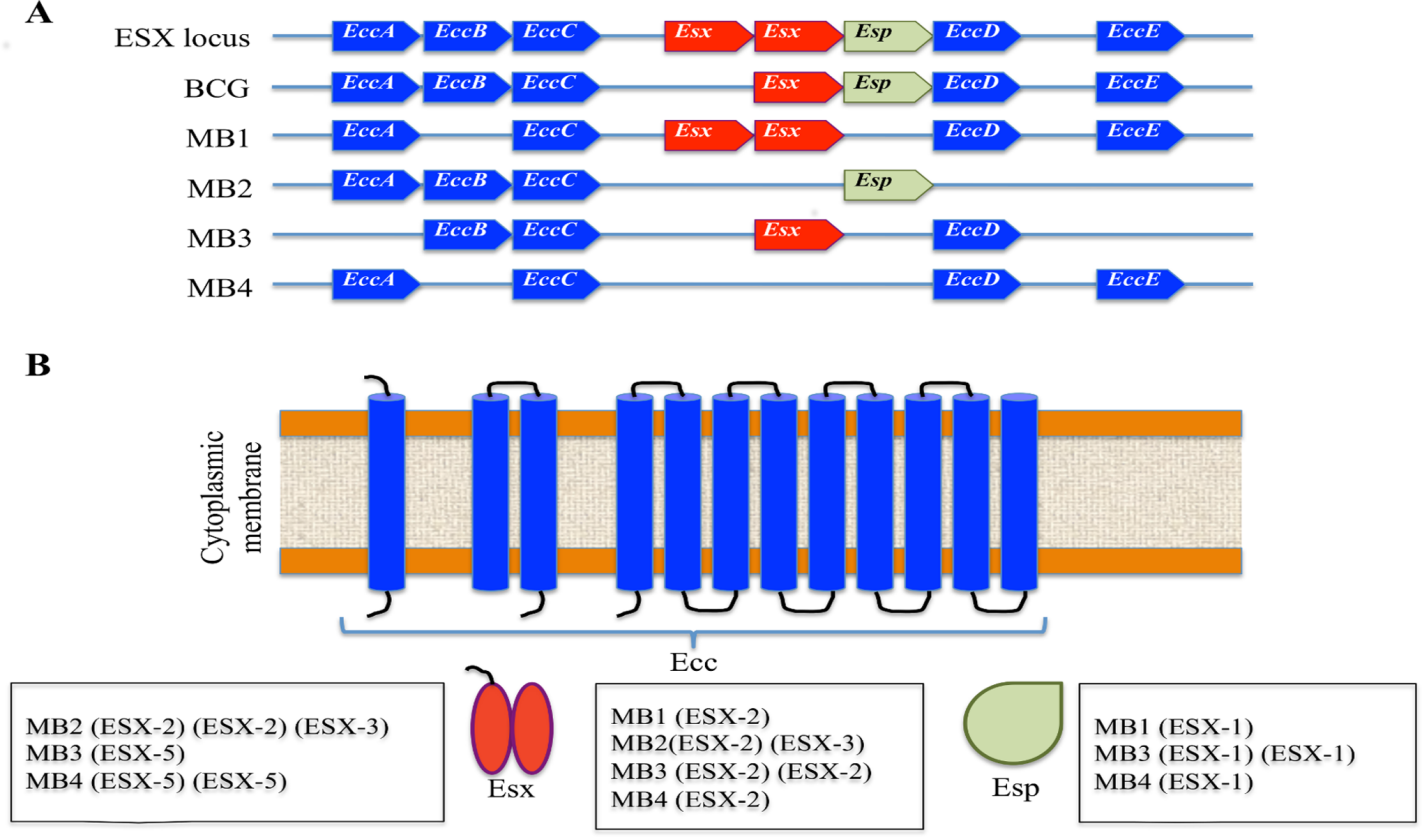
ESX secretion system. (A) Organization of *Ecc*, *Esx* and *Esp* genes in the ESX complete ESX-1 –ESX-5 loci, *M*. *bovis* BCG Pasteur 1173P2 and the genome of MB1-MB4 mycobacteria isolates. (B) Localization of Ecc, Esx and Esp proteins with reference to the ESX loci with deletions in the genome of MB1-MB4 mycobacteria isolates.

ESX-coding genes were annotated in the four genomes analyzed resulting in 53 proteins (S5 Table). Of these, 12 ESX-coding genes were differentially represented due to deletions in the 4 mycobacteria genomes characterized here (Table 2 and Fig 4A).

**Table 2:** Differentially represented ESX-coding genes.

MB1	MB2	MB3	MB4	Description	ESX locus
H8HUI3	NF	H8HUI3	H8HUI3	Putative ESAT-6 like protein EsxD	ESX-2
H8HW13	H8HW13	H8HW13	NF	ESAT-6 like protein 4 (EsxL)	ESX-5
H8I294	H8I294	NF	H8I294	Putative ESAT-6 like protein EsxV	ESX-5
L0PV18	L0PV18	L0PV18	NF	Putative Esat-6 like protein EsxW (ESAT-6 Like Protein 10)	ESX-5
M1J229	NF	M1J229	M1J229	ESAT-6 like protein 11 EsxC	ESX-2
NF	O05449	O05449	NF	VESX-2 secretion system protein EccB2	ESX-2
O05459	NF	NF	O05459	ESX-2 secretion system protein EccE2	ESX-2
O05460	O05460	NF	O05460	ESX-2 secretion system protein EccA2	ESX-2
NF	O69742	NF	NF	ESX-1 secretion-associated protein EspJ	ESX-1
S5EZJ9	S5EZJ9	NF	S5EZJ9	Secretion protein EspD	ESX-1
V2V7U1	NF	V2V7U1	V2V7U1	Secretion protein EccD	ESX-3
V2VSA7	NF	V2VSA7	V2VSA7	EsxH	ESX-3

Uniprot accession numbers are shown for each reference sequence. Abbreviation: NF, not found.

Deletions in the ESX genes included the main components of the ESX system, *Ecc*, *Esx* and *Esp* genes in the ESX-1, ESX-2, ESX-3 and/or ESX-5 loci [50] (Fig 4 and 4B). All *Esp* gene deletions occurred in MB1, MB3 and MB4 isolates and affected the ESX-1 locus (Fig 4A and 4B). EspD of ESX-1 locus has been shown to be critical for virulence in *M*. *tuberculosis* [53], thus suggesting that retention of the *Esp* gene in the *M*. *caprae* MB2 isolate may correlate with its high lesion score when compared to the other sequenced isolates. However, deletion of the *Esp* gene in the MB3 isolate with high lesion score suggested that this locus might be dispensable for virulence in *M*. *bovis*. The attenuation of *M*. *bovis* BCG strains has been associated with deletion of the *EsxA* gene in the ESX-1 locus (Fig 4A) but EsxA or early secreted antigen target protein 6 (ESAT-6) of *M*. *tuberculosis* promotes protective T helper 17 cell responses in a Toll-like receptor-2 dependent manner [17,54]. Recently, *M*. *tuberculosis* EsxH protein of the ESX-3 system was shown to target a host component of the endosomal sorting complex required for transport (ESCRT) to disrupt delivery of mycobacteria to lysosomes [55]. Interestingly, *Esx* gene deletions in MB2-MB4 isolates occurred at ESX-2, ESX-3 or ESX-5 loci but not at the ESX-1 locus (Fig 4A and 4B) suggesting that *Esx* gene deletions in these loci do not affect lesion score produced by *M*. *caprae* and *M*. *bovis*. Immunity conferred by antigen-specific CD4+ T cells is critical for controlling infection with *M*. *tuberculosis* and the ESX-3 locus has been shown to induce maximal responses in the CD4+ T cells screen [50]. This result suggested that deletion in the ESX-3 locus that occurred only in the *M*. *caprae* MB2 isolate might affect the host immune protective response to increase lesion score.

In summary, the analysis of the ESX secretion system in the MB1-MB4 isolates suggested a correlation with MB2 mycobacterial viability or distribution and lesion score. While deletions in the ESX-3 locus of *M*. *caprae* MB2 isolate might affect mycobacterial viability, they could also increase lesion score in conjunction with retention of the *Esp* gene of ESX-1. However, these results suggested that these differences could be host-specific so that the same isolate may behave differently in different hosts.

#### (b) Proteins linking stress response with lipid metabolism

Host stress response induces metabolic changes in mycobacteria that include a switch to the catabolism of host lipids, particularly cholesterol [56]. Recent results have shown that host cells and mycobacteria may interact partly through positive feedback loops, in which responses to the host environment and the digestion of host lipids lead to the production of bacterial immunomodulatory lipids that shape the host environment to increase the availability of host lipids [20]. Following the model recently proposed by Galagan [20] for selected regulatory interactions linking stress responses with changes in lipid metabolism in *M*. *tuberculosis*, genes coding for proteins involved in stress sensing (*phoP*, *whiB3*, *dosR* or *devR*), stress adaptation (*lsr2*, *sigE*, *Rv0081*, *Rv0324*, *Rv3249c*), lipid catabolism (*kstR*, *kstD*, *mcs*, *kshA*) and lipid production (*Rv1353c*, *Rv1776c*, *pks2*, *pks3*, *drrA*, *drrB*, *drrC*, *mmpL7*) were selected for analysis (S3 Table).

All genes but the methylcitrate synthase gene (*mcs*) were found in all genomes analyzed. The *mcs* gene was not found in any of the MB1-MB4 isolates analyzed nor in the *M*. *bovis* reference genome, thus suggesting a deletion of this gene in *M*. *bovis/M*. *caprae*. The *mcs* gene is important for lipid catabolism [20]. Furthermore, mycobacterial metabolism of propionyl-CoA is also important because accumulated propionate as well as MCS/MCD-generated propionate metabolites are toxic and exert a dominant inhibitory effect on bacterial growth [57]. We speculate that this deletion increases *M*. *bovis* susceptibility to lipid deprivation, correlating with the TB-resistant phenotype associated with higher methylmalonyl CoA mutase (MUT) levels in wild boar [56, 58–60]. High MUT levels will reduce host cholesterol and thus put an additional pressure on *M*. *bovis* survival in this species [56].

**Fig 5:**
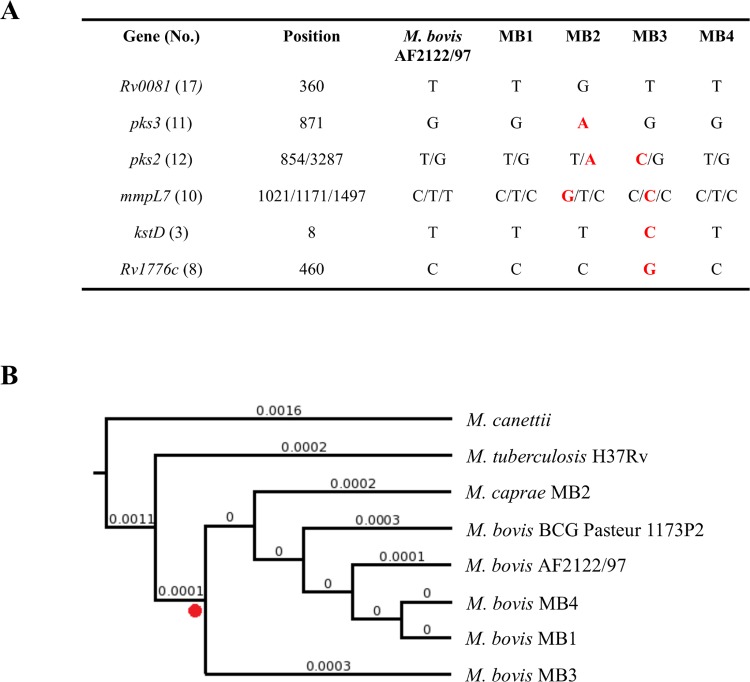
Genes coding for the proteins linking stress response with lipid metabolism. (A) SNPs detected in mycobacteria isolates MB1-MB4 with *M*. *bovis* AF2122/97 reference genome sequence. Non-synonymous substitutions are shown in red. Gene No. refers to row number in S3 Table. (B) Multilocus sequence analysis. The nucleotide sequences of the selected genes were concatenated and aligned to construct a maximum likelihood phylogenetic tree. Numbers of internal branches represent genetic distances. The reliability for the internal branches was assessed using the approximate likelihood ratio test (aLRT–SH-Like). The clade of *M*. *bovis/M*. *caprae* (red dot) is supported by an aLRT–SH-Like value of 87. *M*. *canettii* was used as outgroup. The image was built using EvolView.

The multilocus sequence analysis was conducted using 18 genes coding for the proteins linking stress response with lipid metabolism (S3 Table). SNPs were found in 6 genes only with few non-synonymous substitutions (Fig 5A). Furthermore, no evidence was found for a positive or negative selection (P>0.05) in the codons where SNPs were identified. Interestingly, the highest number of non-synonymous substitutions was found in the MB2 and MB3 isolates with high lesion score (Fig 5A). The phylogenetic analysis corroborated the results at genome level (Fig 2A) with a strong support for the *M*. *bovis/M*. *caprae* clade (Fig 5B).

These results showed that genes coding for the proteins linking stress response with lipid metabolism are highly conserved in *M*. *bovis/M*.*caprae* and suggested that deletion of the *mcs* gene may increase susceptibility to lipid deprivation in these mycobacteria. However, some of the non-synonymous substitutions found in MB2 and MB3 may provide an advantage resulting in higher lesion score for these isolates.

#### (c) Host T cell epitopes of mycobacteria

The experimentally confirmed human T cell epitopes of *M*. *tuberculosis* [17] were analyzed in the MB1-MB4 genomes. These epitopes are hyperconserved in *M*. *tuberculosis* and *M*. *africanum* strains consistent with strong purifying selection acting on these epitopes. As discussed by Comas et al. [17], MTBC might benefit from recognition by human T cells because this essential response for host survival may be necessary for mycobacteria to establish latent infection. Thus, T lymphocyte recognition is an important factor in hyperconservation of these sequences and hence other structural or functional constraints are unlikely to fully account for the lack of sequence variation in these domains.

**Fig 6:**
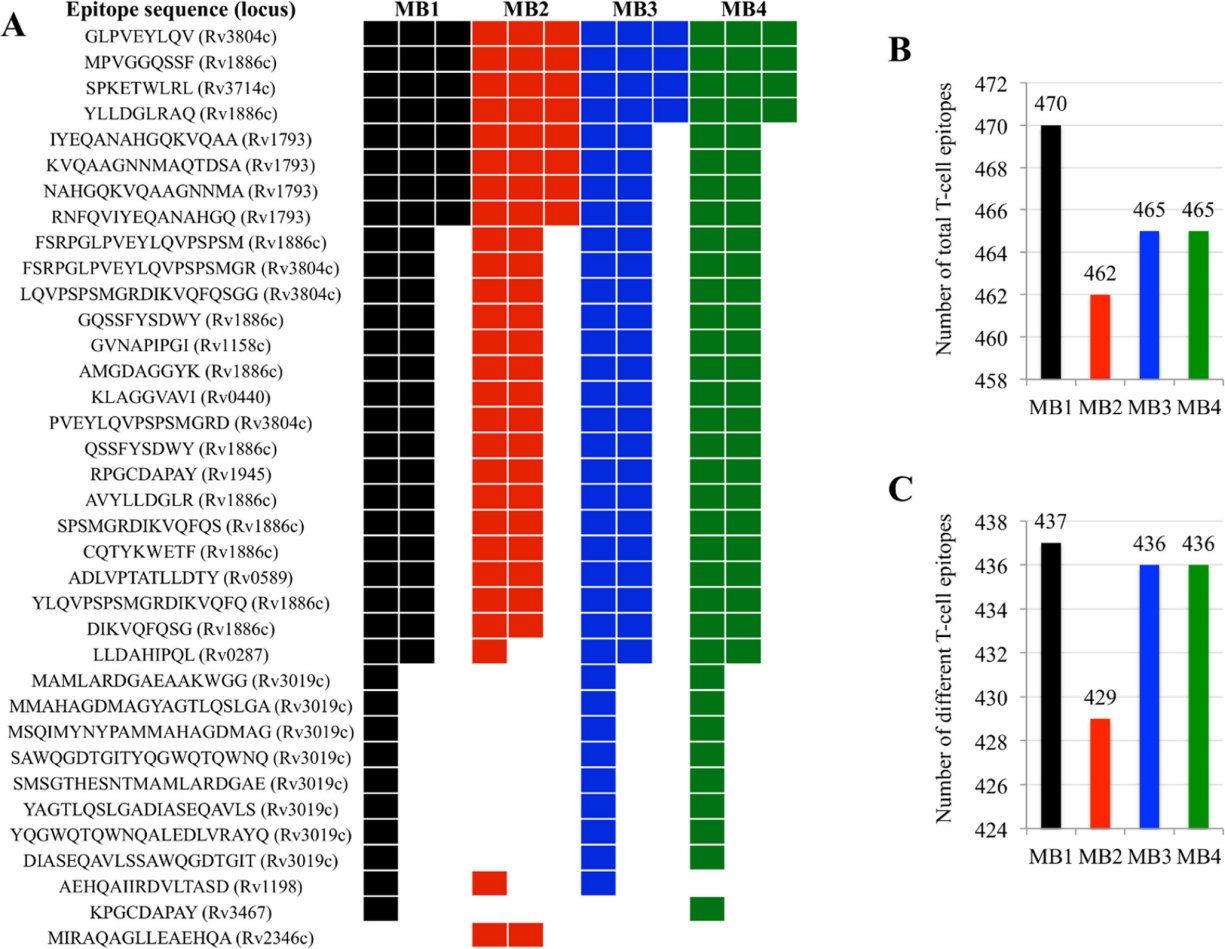
Host T cell epitopes of mycobacteria. (A) Organization of the repeated/deleted epitopes in the different sequenced mycobacteria isolates. The locus name of the antigen in the H37Rv genome for T-cell epitope containing antigens [17] is shown in parenthesis after epitope sequence. The protein annotation according to the Immune Epitope Database (IEDB; http://www.iedb.org) can be found in S4 Table. Each column represents epitope repeat or deletion. (B) Total number of T cell epitopes including repetitions identified on each mycobacteria. (C) Number of different T cell epitopes identified on each mycobacteria.

Of the 491 epitopes included in the analysis [17], 438 were identified in the MB1-MB4 isolates sequenced here with 100% sequence identity to *M*. *tuberculosis* sequences (S6 Table). These results extended the hyperconservation of these epitopes to *M*. *bovis/M*. *caprae* and suggested that similar mechanisms may function in other species of the MTBC. However, several specific patterns of epitope repetitions and deletions were found in the studied isolates (Fig 6A). Interestingly, MB2 was the isolate with the lowest number of both total (Fig 6B) and different (Fig 6C) T cell epitopes, correlating with the lowest distribution of this isolate (Table 1) thus providing additional support for the role of the T cell response in mycobacterial transmission. One limitation of this analysis is the fact that the T cell epitopes used are confirmed in humans but not in other hosts susceptible to *M*. *bovis/M caprae* infection. However, the approach used in this study provided an initial assessment of the presence and possible role of the host T cell epitopes in *M*. *bovis/M*. *caprae* infection and transmission.

#### (d) Antigens

**Fig 7:**
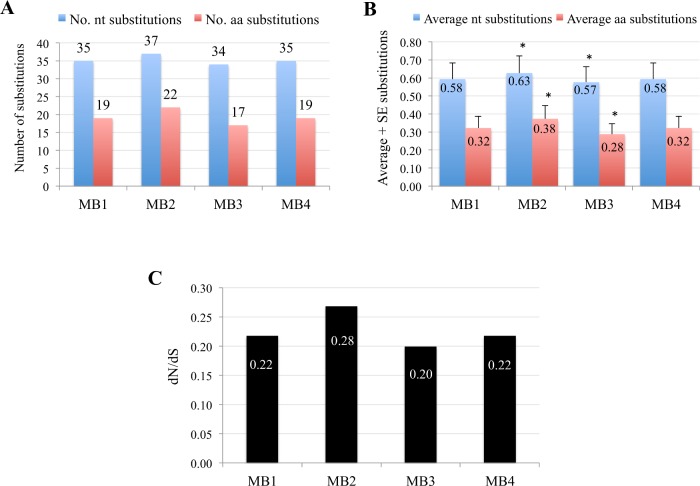
Nucleotide substitution rate analysis in antigen-coding genes. (A) The total number and (B) the average + standard error (SE) of nucleotide (nt) and amino acid (aa) substitutions were calculated in 81 antigen-coding genes conserved in MB1-MB4 isolates using *M*. *tuberculosis* H37Rv as a reference. Average values were compared between isolated using a χ2 test (*P<0.0001). (C) The dN/dS ratio was determined on the basis of the number of non-redundant synonymous and non-synonymous changes using the SLAC algorithm and a two-tailed extended binomial distribution was used to assess significance of the algorithm (P<0.05).

The 81 antigens conserved in MB1-MB4 isolates containing the confirmed human T cell epitopes of *M*. *tuberculosis* [17] or inducing an antibody response against inactivated *M*. *bovis* [8] were selected for further characterization using the number of nucleotide and amino acid substitutions in each of the isolates (S4 Table). Of them, 39 were conserved 100% at the nucleotide sequence and 33 showed polymorphisms when compared to the *M*. *tuberculosis* H37Rv sequence (S4 Table). The results showed that as in *M*. *tuberculosis* [17] the antigen-coding genes were evolutionarily hyperconserved with a low number of nucleotide and amino acid substitutions (Fig 7A). However, the average number of substitutions at both nucleotide and amino acid levels were significantly (P<0.0001) higher and lower for MB2 and MB3 isolates, respectively with no differences between MB1 and MB4 isolates (Fig 7B). However, the values for dN/dS ratios were higher for *M*. *tuberculosis* (0.5; [17]) than for *M*. *bovis/M*. *caprae* (0.20–0.28; Fig 7C) thus suggesting that these genes are under a strong purifying selection in these species.

As reported for *M*. *tuberculosis* [17], despite high sequence conservation in these antigens, sequencing revealed a subset of antigens that did show variation (Table 3). These antigens included proteins that are involved in bacterial infection (MCE-family protein MCE2a, Fibronectin attachment protein; [61,62]), induction of host immune response to infection (ESAT-6 like protein 4 EsxL; [17,54]), clustered regularly interspaced short palindromic repeats (CRISPRs) bacterial adaptive immunity against mobile genetic elements (CRISPR-associated protein Cas10/Csm1; [63]) and recombination and DNA repair (ATP-dependent DNA helicase; [64]) and thus may warrant further study in the context of vaccine discovery.

**Table 3:** Antigens with high sequence variation.

Sequence ID (H37Rv)	Description	Number of amino acids substitutions			
		MB1	MB2	MB3	MB4
Rv0589	MCE-family protein MCE2a	3	2	2	3
Rv1198	ESAT-6 like protein 4 EsxL	2	1	2	2
Rv1860	Fibronectin attachment protein FAP-B	1	2	1	1
Rv2823c	CRISPR-associated protein Cas10/Csm1	2	2	0	2
Rv3296	ATP-dependent helicase	2	1	3	2

Protein sequence diversity among all isolates was determined in comparison to *M*. *tuberculosis* H37Rv. Antigens selected showed an average amino acid diversity > 1.

#### (e) Peptidoglycan assembly protein ponA1 (Rv0050)

The *M*. *tuberculosis* hypervariable locus Rv0050 coding for PonA1 was targeted for analysis due to its role in glycan chain assembly and peptide cross-linking of bacterial peptidoglycan. PonA1 is a member of a family of penicillin binding proteins that may be essential to mycobacterial survival [65,66]. Although known to be hypervariable among bacteria [67,68], Rv0050 was very conserved (>99%; 698/704) among all *M*. *bovis* strains. A cluster of three non-synonymous mutations at the N terminus of the protein was identified for MB1 to MB4 compared to *M*. *tuberculosis* H37Rv (S3A Fig). The analysis of the N terminus region of PonA1 revealed a putative inner membrane domain and a cleavage site between amino acids 31 and 32 (S3A and S3B Fig), which exhibited homology to *M*. *leprae* LprE [46]. The isolate with high lesion score, MB3, had two mutations in the putative inner membrane domain P24L and T29M. The other isolate with high lesion score, MB2 (*M*. *caprae*), did not contain the mutation P24L but had instead mutation T27I in the putative inner membrane domain closer to the putative cleavage site located between amino acids 31 and 32. Mutations P24L, T27I, and T29M all exchanged small (P or T) residues with very hydrophobic residues (L, I, and M) that may favor insertion/delivery of PonA1 to the bacterial membrane. All three mutations may also change the way PonA1 may be cleaved as substitutions upstream of the cleavage site have a bigger penalty in mycobacteria [69].

MB1 and MB4 contained only mutation P24L that is present in all *M*. *bovis* isolates infecting animals. Only *M*. *bovis* human isolate BZ3115 contained mutation P24L (S3A Fig) while all other human isolates of *M*. *bovis* had a sequence identical to strains of *M*. *tuberculosis* in the complex (wild type P at position 24; S3A Fig). The selective pressure for mutations on PonA1 may affect uniquely strains of *M*. *bovis* infecting animals, as all other members of the MTBC including strains of *M*. *bovis* infecting humans have identical wild type sequences. A possible ancestry [20] in strains of *M*. *bovis* infecting humans and *M*. *tuberculosis* is also supported through strain BZ3115 PonA1 sequence (S3A Fig).

To confirm some of the SNPs identified in the mycobacteria genomes sequenced in this study, targeted PCR and sequence analysis were conducted for Rv0050 (ponA1), Rv0589 (MCE2a), Rv1198 (ESAT-6) and Rv1860 (FAP-B) loci with potential interest for TB disease risk assessment and control. The results corroborated the predictions of the genome sequencing for the MB1-MB4 isolates included in the study, thus providing additional support for the results presented and discussed in the paper (S4 Fig).

**Fig 8:**
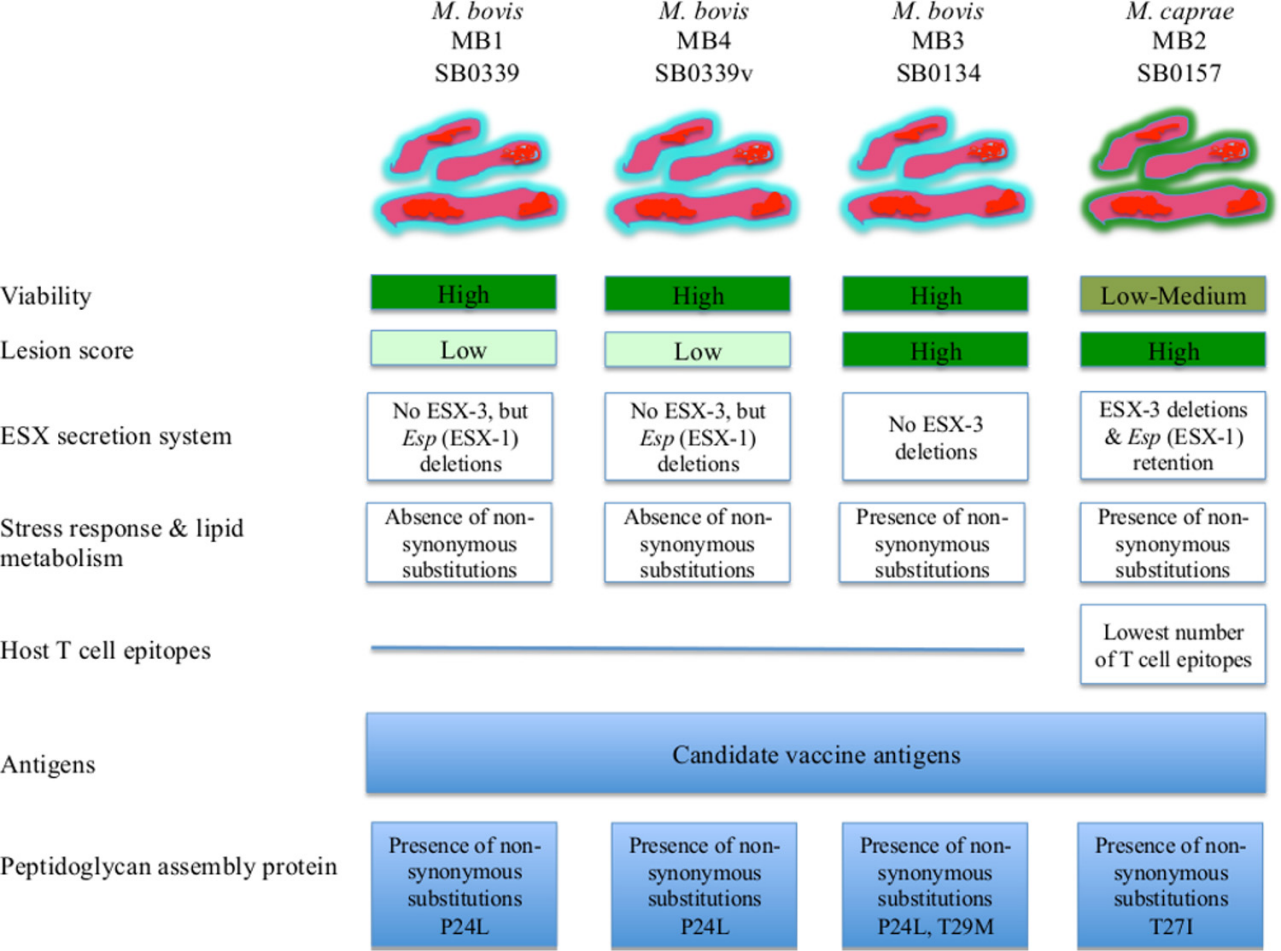
Summary of the results of strategic protein-targeted comparative analysis. Protein-targeted comparative analysis was conducted for (a) the ESX or type VII secretion system, (b) proteins linking stress response with lipid metabolism, (c) host T cell epitopes of mycobacteria, (d) antigens and (e) penicillin binding protein to define possible correlates with bacterial distribution and lesion score and to identify genetic markers and candidate antigens for TB disease risk assessment and control.

In summary, this is the first comparative genomics study of field isolates of *M*. *bovis* including for the first time *M*. *caprae*. Three *M*. *bovis* (MB1, MB3, MB4) and one *M*. *caprae* (MB2) isolates with different distribution and lesion score were selected for genome sequencing and annotation (Fig 8). Comparative genomics showed that as previously reported for *M*. *tuberculosis*, sequential chromosomal nucleotide substitutions were the main driver of the *M*. *bovis* genome evolution. The phylogenetic analysis provided a strong support for the *M*. *bovis/M*. *caprae* clade, but supported *M*. *caprae* as a separate species. The comparison of the MB1 and MB4 isolates revealed differences in genome sequence, including gene families that are important for bacterial infection and transmission, thus highlighting differences with functional implications between MTBC isolates otherwise classified with the same spoligotype. Protein-targeted comparative analysis was conducted for (a) the ESX or type VII secretion system, (b) proteins linking stress response with lipid metabolism, (c) host T cell epitopes of mycobacteria, (d) antigens and (e) penicillin binding protein to define possible correlates with bacterial distribution and lesion score. The analysis of the ESX secretion systems provided possible correlates for MB2 low-medium distribution (ESX-3 deletions) and high lesion score (retention of ESX-1 *Esp*) but suggested that these differences could be host-specific so that the same isolate might behave differently in different hosts (Fig 8). These results showed that genes coding for the proteins linking stress response with lipid metabolism are highly conserved in *M*. *bovis/M*.*caprae* and suggested that deletion of the *mcs* gene may increase susceptibility to lipid deprivation in these mycobacteria. However, some of the non-synonymous substitutions found in MB2 and MB3 may provide an advantage resulting in higher lesion score for these isolates (Fig 8). MB2 was the isolate with the lowest number of T cell epitopes, correlating with the lowest distribution of this isolate if we consider that recognition by T cells could be essential to establish mycobacterial latent infection [17] (Fig 8). The genes coding for mycobacterial antigens were highly conserved and under a strong purifying selection in *M*. *bovis/M*.*caprae*, thus extending previous results in *M*. *tuberculosis*. The polymorphisms provided new candidate vaccine antigens. The comparison of PonA1 sequences among MB1-MB4 isolates suggested a possible correlation with their phenotypic variation with possible implications for TB disease risk assessment and control (Fig 8). The accumulation of mutations at the putative inner membrane protein domain suggested that the retention of mutations that affected delivery/insertion or cleavage of ponA1 at the membrane might have been the result of the forces driving selection. Because of the role of PonA1 in cell wall assembly, the described mutations may have a link to environmental selective pressures such as transmission and pathogenesis. These results identified new genetic markers and candidate vaccine antigens that warrant further study providing additional experimental data to develop tools to evaluate risks for TB disease caused by *M*. *bovis /M*.*caprae* and for TB control in humans and animals.

## Supporting Information

S1 Fig Frequency (%) of reported spoligotypes in livestock farms and wild ungulate populations in Ciudad Real province (N = 900 MTBC isolates). (DOCX)

S2 Fig Genome sequence comparison between mycobacteria isolates using Differences program. (DOCX)

S3 Fig Peptidoglycan assembly protein PonA1 sequence analysis. (DOCX)

S4 Fig PCR and sequence analysis of selected loci with predicted SNPs in MB1-MB4 genomes. (DOCX)

S1 Table Genome sequence assembly using Velvet with k-value = 97. (DOCX)

S2 Table Genome annotation for MB1-MB4 isolates. (XLS)

S3 Table Stress response and lipid metabolism proteins identified in the MB1-MB4 genomes. (XLSX)

S4 Table Antigens selected for analysis in the MB1-MB4 isolates. (DOCX)

S5 Table ESX proteins identified in the MB1-MB4 genomes. (DOCX)

S6 Table T-cell epitopes identified in the MB1-MB4 genomes. (XLSX)
