# Supplementary material for: Comparative Genomics of Field Isolates of Mycobacterium bovis and M. caprae Provides Evidence for Possible Correlates with Bacterial Viability and Virulence
Source: PLoS Negl Trop Dis. 2015 Nov 19;9(11):e0004232. doi: 10.1371/journal.pntd.0004232 (PMC4652870; doi:10.1371/journal.pntd.0004232)
Supplement: S1 Fig — (DOCX) [file pntd.0004232.s001.docx]

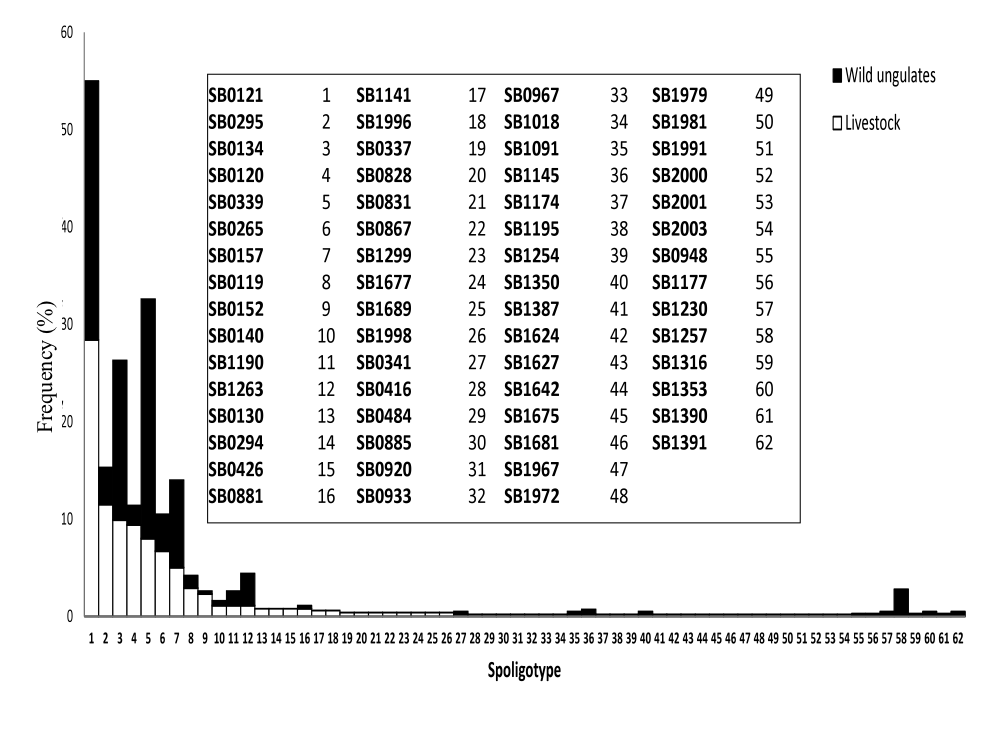
**S1 Figure.** Frequency (%) of reported spoligotypes in livestock farms and wild ungulate populations in Ciudad Real province (N=900 MTBC isolates). The numerical codes at the bottom of the bars correspond with spoligotypes as indicated in the panel inside the figure (spoligotypes are decreasingly ranked in the table and figure according to the frequency they appear in livestock).
