# Supplementary material for: Comparative Genomics of Field Isolates of Mycobacterium bovis and M. caprae Provides Evidence for Possible Correlates with Bacterial Viability and Virulence
Source: PLoS Negl Trop Dis. 2015 Nov 19;9(11):e0004232. doi: 10.1371/journal.pntd.0004232 (PMC4652870; doi:10.1371/journal.pntd.0004232)
Supplement: S2 Fig — (DOCX) [file pntd.0004232.s002.docx]

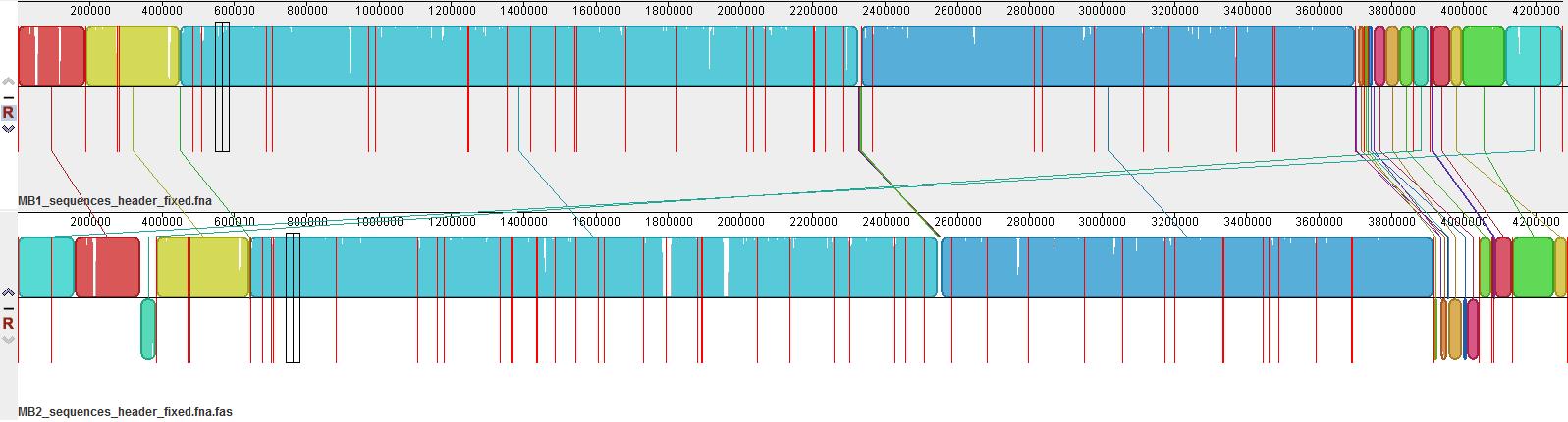


MB1 (upper) vs. MB2 (lower)


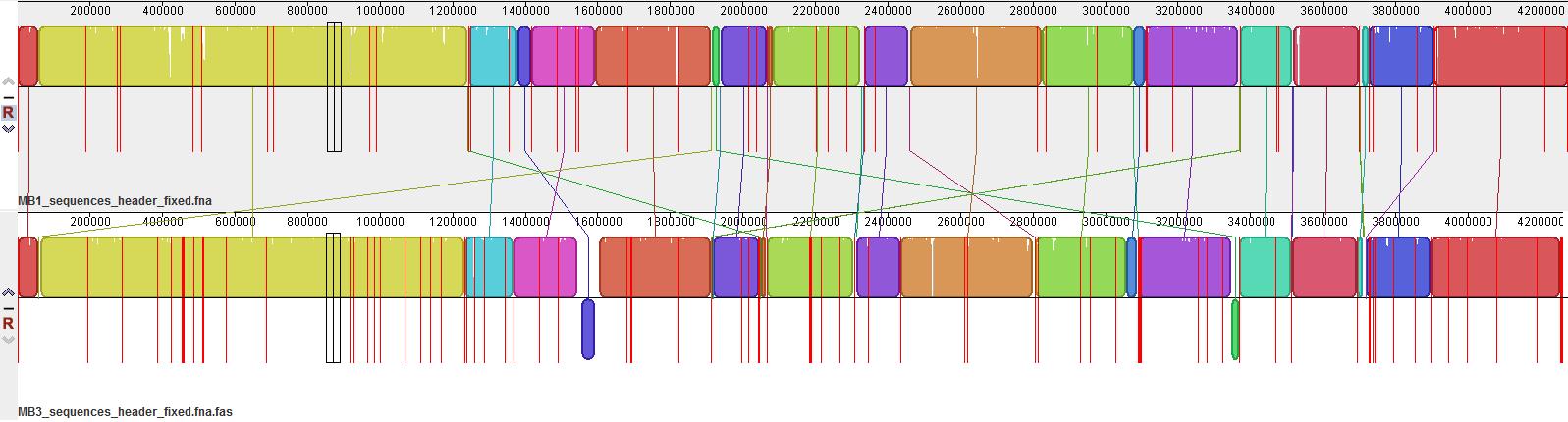


MB1 (upper) vs. MB3 (lower)


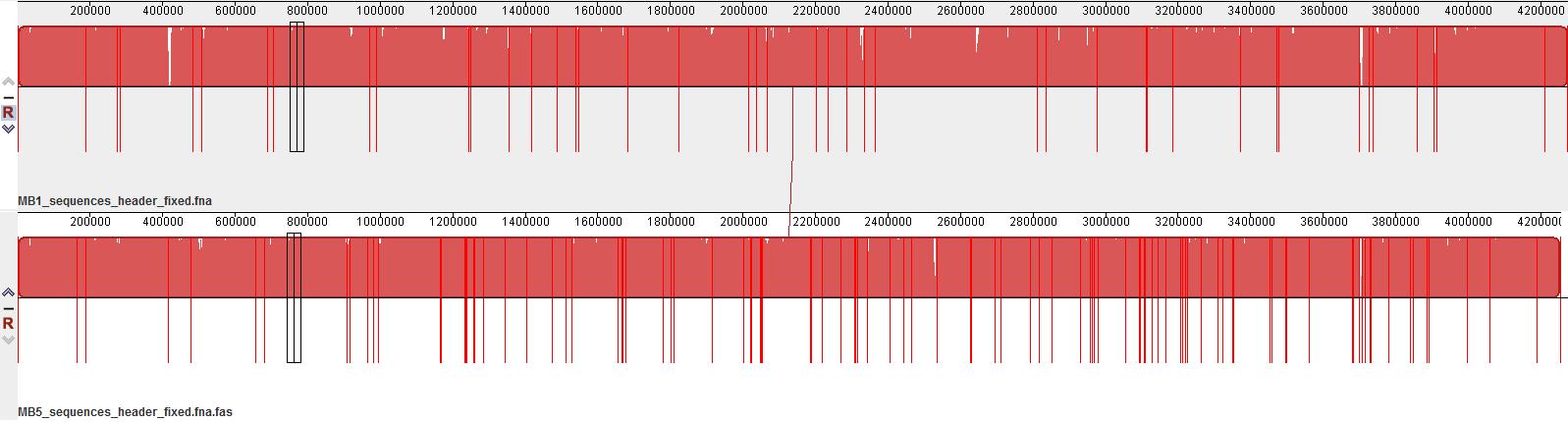


MB1 (upper) vs. MB4 (lower)


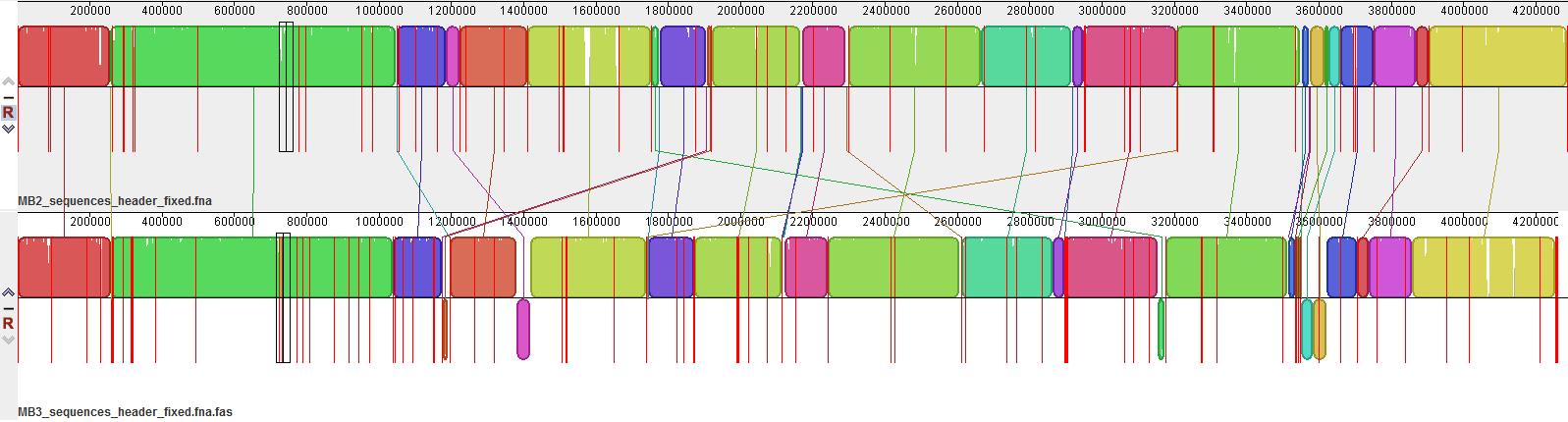


MB2 (upper) vs. MB3 (lower)


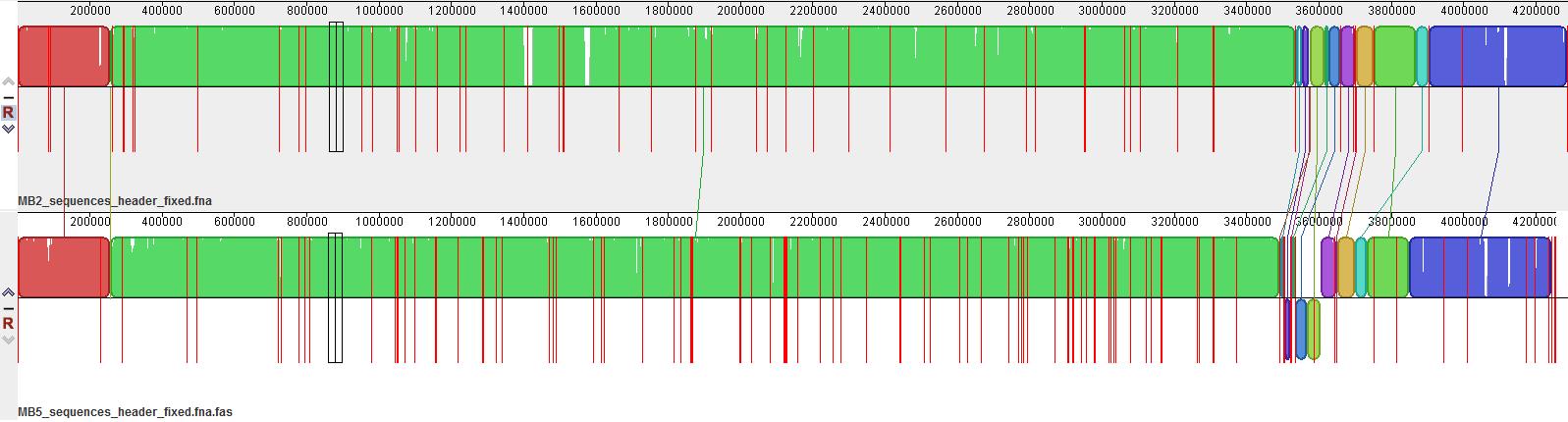


MB2 (upper) vs. MB4 (lower)


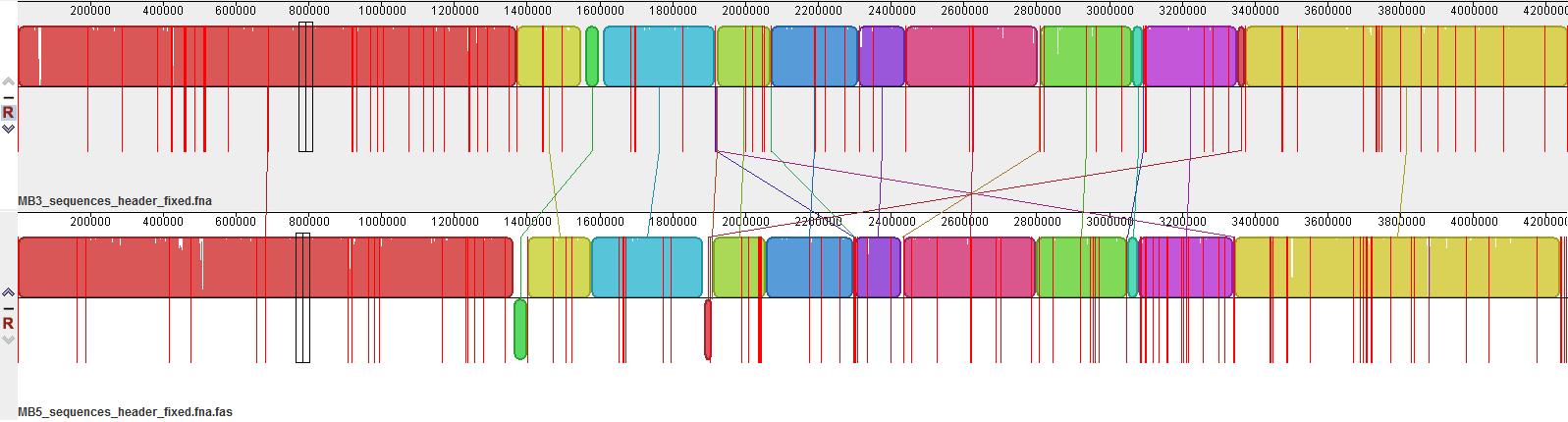


MB3 (upper) vs. MB4 (lower)

**S2 Figure.** Genome sequence comparison between mycobacteria isolates using Differences program.
