# Supplementary material for: Comparative Genomics of Field Isolates of Mycobacterium bovis and M. caprae Provides Evidence for Possible Correlates with Bacterial Viability and Virulence
Source: PLoS Negl Trop Dis. 2015 Nov 19;9(11):e0004232. doi: 10.1371/journal.pntd.0004232 (PMC4652870; doi:10.1371/journal.pntd.0004232)
Supplement: S3 Fig — (DOCX) [file pntd.0004232.s003.docx]

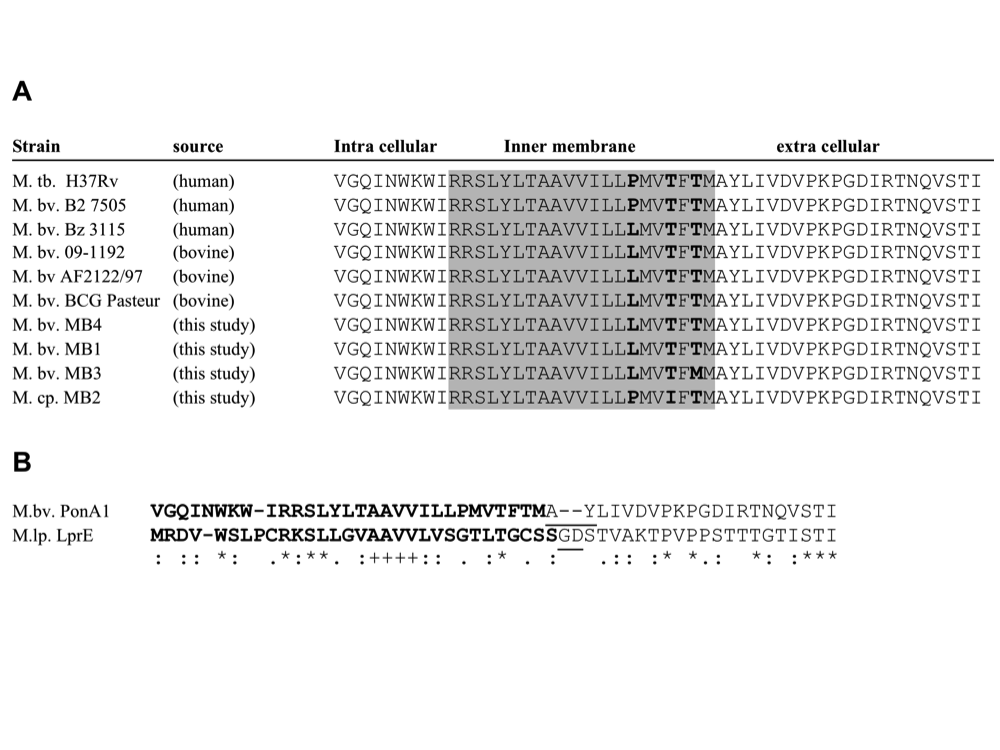


S3 Figure. Peptidoglycan assembly protein PonA1 sequence analysis. (A) PonA1 sequence analysis. Bold type letters indicate residues that are mutated within the MTBC. Putative inner membrane residues are grayed. (B) Signal peptide prediction and alignment to its closest homologous sequences in mycobacteria. Bold type letters indicate the putative signal peptide sequence. Putative cleavage sites are underlined. Asterisks indicate identical residues, while colons and periods indicate conserved and semiconserved residues, respectively. Abbreviations: M. tb., *M. tuberculosis*; M.bv., M. bovis; M. cp., *M. caprae*., M.lp., M. leprae., LprE, signal peptide database LPRE_MYCLE.
