# Supplementary material for: Comparative Genomics of Field Isolates of Mycobacterium bovis and M. caprae Provides Evidence for Possible Correlates with Bacterial Viability and Virulence
Source: PLoS Negl Trop Dis. 2015 Nov 19;9(11):e0004232. doi: 10.1371/journal.pntd.0004232 (PMC4652870; doi:10.1371/journal.pntd.0004232)
Supplement: S4 Fig — (DOCX) [file pntd.0004232.s004.docx]

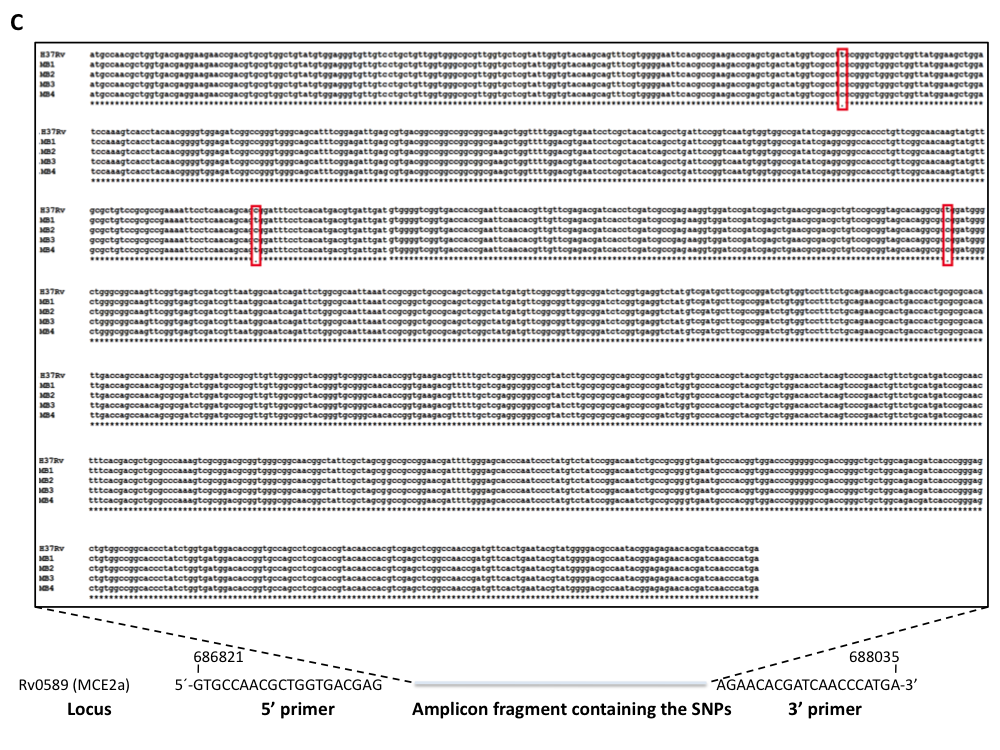

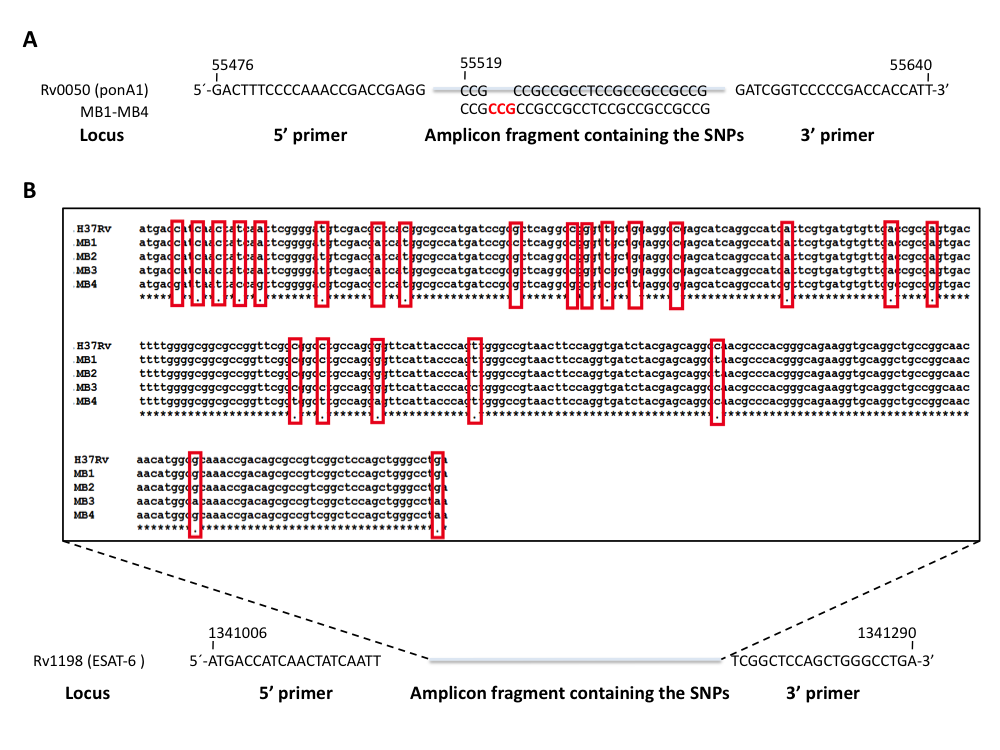


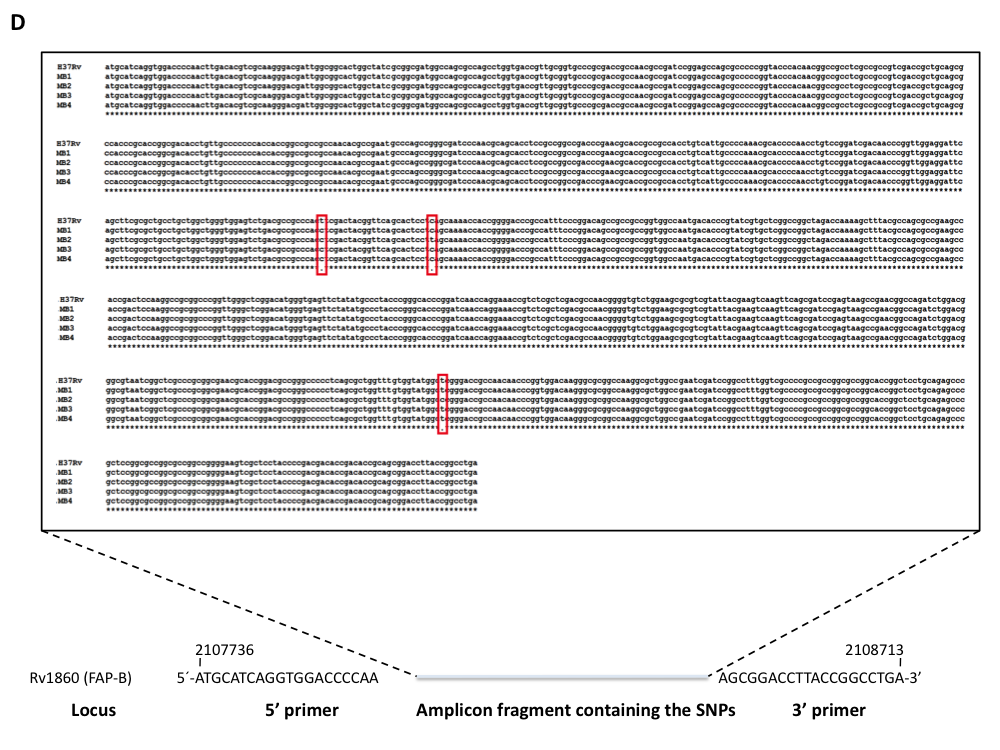


**S4 Figure.** PCR and sequence analysis of selected loci with predicted SNPs in MB1-MB4 genomes. To confirm selected SNPs identified in the mycobacteria genomes sequenced in this study, sequence-specific oligonucleotide primers were design for PCR and sequence analysis of the (A) Rv0050 (ponA1), (B) Rv1198 (ESAT-6), (C) Rv0589 (MCE2a), and (D) Rv1860 (FAP-B) loci with potential interest for TB disease risk assessment and control. Nucleotide position numbers are referred to reference genomes *M. bovis* AF2122/97 (BX248333.1) (Rv0050, mmpL7) or *M. tuberculosis* H37Rv (AL123456.3) (Rv0589, Rv1198, Rv1860). SNPs in the MB1-MB4 genomes with respect to reference genomes are shown in red.
