# Supplementary material for: Comparative Genomics of Field Isolates of Mycobacterium bovis and M. caprae Provides Evidence for Possible Correlates with Bacterial Viability and Virulence
Source: PLoS Negl Trop Dis. 2015 Nov 19;9(11):e0004232. doi: 10.1371/journal.pntd.0004232 (PMC4652870; doi:10.1371/journal.pntd.0004232)
Supplement: S1 Table — (DOCX) [file pntd.0004232.s005.docx]

**S1 Table.** Genome sequence assembly using Velvet with k-value = 97.

|  | **Mycobacteria isolate** | | | |
| --- | --- | --- | --- | --- |
| **Sequence assembly** | **MB4** | **MB3** | **MB2** | **MB1** |
| No. contigs  (≥ 0 bp) | 118 | 94 | 59 | 49 |
| No. contigs  (≥ 1000 bp) | 103 | 87 | 56 | 46 |
| Total length (≥ 0 bp) | 4255612 | 4259788 | 4288871 | 4275214 |
| Total length (≥ 1000 bp) | 4248060 | 4257158 | 4288022 | 4273544 |
| No. contigs | 109 | 88 | 56 | 47 |
| Total length (bp) | 4252126 | 4257680 | 4288022 | 4274471 |
| Largest contig (bp) | 229115 | 230159 | 291973 | 447815 |
| GC (%) | 65.59 | 65.58 | 65.58 | 65.60 |
| N50 | 81862 | 93843 | 120976 | 188047 |
| N75 | 46622 | 52415 | 88780 | 123412 |
| L50 | 16 | 15 | 13 | 9 |
| L75 | 33 | 30 | 23 | 16 |
| No. N's per 100 kbp | 0.00 | 224.37 | 245.26 | 292.64 |

N50 and N75 are the contig lengths such that using longer or equal length contigs produces 50% or 75%, respectively of the bases of the reference genome. This metric could be computed only if a reference genome is given. L50 and L75 are the numbers of contigs longer or equal to corresponding N-metrics. In other words, L50, for example, is the minimal number of contigs that cover at least 50% of total genome length. Definitions were taken from http://sourceforge.net/p/quast/wiki/Manual%201.2/.
