# Supplementary material for: Comparative Genomics of Field Isolates of Mycobacterium bovis and M. caprae Provides Evidence for Possible Correlates with Bacterial Viability and Virulence
Source: PLoS Negl Trop Dis. 2015 Nov 19;9(11):e0004232. doi: 10.1371/journal.pntd.0004232 (PMC4652870; doi:10.1371/journal.pntd.0004232)
Supplement: S4 Table — (DOCX) [file pntd.0004232.s008.docx]

### **S4 Table.** Antigens selected for analysis in the MB1-MB4 isolates.

**Accession ID Immune Epitope Database (IEDB) annotation**

Rv0125 * PROBABLE SERINE PROTEASE PEPA (SERINE PROTEINASE) (MTB32A)

Rv0129c* Antigen 85-C precursor

Rv0200 * orfH-200

Rv0203 * hypothetical protein MT0213

Rv0222 enoyl-CoA hydratase

Rv0287 * Hypothetical protein esxG

Rv0288 * ESAT-6-like protein esxH

Rv0309** hypothetical protein MT0322

Rv0341* ISONIAZID INDUCTIBLE GENE PROTEIN INIB

Rv0440 * 60 KDA CHAPERONIN 2 GROEL2 (PROTEIN CPN60-2)

Rv0589 ** MCE-FAMILY PROTEIN MCE2A

Rv0667 ** DNA-directed RNA polymerase subunit beta

Rv0670 ** endonuclease IV

Rv0934 ** Phosphate-binding protein pstS 1 precursor

Rv1157c* hypothetical protein MT1193

Rv1158c CONSERVED HYPOTHETICAL ALA-, PRO-RICH PROTEIN

Rv1174c* LOW MOLECULAR WEIGHT T-CELL ANTIGEN TB8.4

Rv1184c** hypothetical protein MT1221

Rv1198 ** PUTATIVE ESAT-6 LIKE PROTEIN ESXL (ESAT-6 LIKE PROTEIN 4)

Rv1242 * hypothetical protein MT1280

Rv1255c** PROBABLE TRANSCRIPTIONAL REGULATORY PROTEIN

Rv1256c PROBA BLE CYTOCHROME P450 130 CYP130

Rv1280c* PROBABLE PERIPLASMIC OLIGOPEPTIDE-BINDING LIPOPROTEIN OPPA

Rv1291c CONSERVED HYPOTHETICAL SECRETED PROTEIN

Rv1461** hypothetical protein Rv1461

Rv1623c** Cytochrome BD-I oxidase subunit I

Rv1641 * translation initiation factor IF-3

Rv1694 ** CYTOTOXIN|HAEMOLYSIN HOMOLOGUE TLYA

Rv1793 * ESAT-6-like protein esxN

Rv1818c PE_PGRS 33

Rv1860 ** fibronectin attachment protein

Rv1886c* Antigen 85-B precursor

Rv1926c* IMMUNOGENIC PROTEIN MPT63 (ANTIGEN MPT63/MPB63)

Rv1945** hypothetical protein Rv1945

Rv1977 hypothetical protein Rv1977

Rv1980c* Immunogenic protein MPT64 precursor

Rv1985c** chromosome replication initiation inhibitor protein

Rv1986 * PROBABLE CONSERVED INTEGRAL MEMBRANE PROTEIN

Rv1987 * POSSIBLE CHITINASE

Rv2031c* 14 kDa antigen

Rv2182c* 1-acylglycerol-3-phosphate O-acyltransferase

Rv2190c* NLP/P60 family protein

Rv2220 * Glutamine synthetase 1

Rv2223c* proteinase

Rv2290 ** lipoprotein lppO

Rv2346c Putative ESAT-6-like protein 6

Rv2351c mtp40 protein - Mycobacterium tuberculosis

Rv2476c** PROBABLE NAD-DEPENDENT GLUTAMATE DEHYDROGENASE GDH

Rv2666 PROBABLE TRANSPOSASE FOR INSERTION SEQUENCE ELEMENT IS1081

Rv2715 ** Uncharacterized protein Rv2715/MT2788

Rv2780* Alanine dehydrogenase

Rv2823c** hypothetical protein Rv2823c: CRISPR-associated protein Cas10/Csm1

Rv2875 * Immunogenic protein MPB70 precursor

Rv2878c** Soluble secreted antigen MPT53 precursor

Rv2903c Probable signal peptidase I

Rv2945c* Putative lipoprotein lppX precursor

Rv3017c* ESAT-6-like protein esxQ

Rv3019c ESAT-6-like protein esxR

Rv3106 ** ferredoxin--NADP reductase

Rv3201c* helicase, UvrD/Rep family

Rv3207c** conserved hypothetical protein, truncation

Rv3296 ** ATP-dependent helicase, putative

Rv3333c** hypothetical protein MT3437

Rv3378c** hypothetical protein Rv3378c

Rv3418c* 10 kDa chaperonin

Rv3467 hypothetical protein Rv3467

Rv3497c* MCE-FAMILY PROTEIN MCE4C

Rv3619c antigen Mtb9.9B

Rv3689 ** PROBABLE CONSERVED TRANSMEMBRANE PROTEIN

Rv3714c** hypothetical protein Rv3714c

Rv3763 * Lipoprotein lpqH precursor

Rv3803c* MPT51/MPB51 antigen precursor

Rv3804c** Antigen 85-A precursor

Rv3812 * hypothetical glycine-rich protein Rv3812

Rv3846 ** Superoxide dismutase

Rv3871* FtsK/SpoIIIE family protein

Rv3873 ** PPE FAMILY PROTEIN

Rv3874 * 10 KDA CULTURE FILTRATE ANTIGEN ESXB (LHP) (CFP10)

Rv3875 ** 6 kDa early secretory antigenic target

Rv3878 ** CONSERVED HYPOTHETICAL ALANINE RICH PROTEIN

Rv3879c** hypothetical protein MT3993

Rv1436 * glyceraldehyde-3-phosphate dehydrogenase (GAPDH)

Rv3045** NADP-dependent alcohol dehydrogenase C (NADPAD)

Rv2873 * MPB83, Fasciclin

Rv2623 * universal stress protein Rv2623/MT2698

The first column indicates the locus name of the antigen in the H37Rv genome for T-cell epitope containing antigens [17] and for *M. bovis* proteins inducing antibody response in wild boar (last 4 entries; [8]). Asterisks mark the sequences included in the study and conserved in MB1-MB4 isolates. One asterisk indicates 100% conserved sequences at both nucleotide and amino acid levels in all MB1-MB4 isolates while two asterisks indicate the sequences with polymorphisms when compared to *M. tuberculosis* H37Rv. The second column indicates protein annotation according to the Immune Epitope Database (IEDB; http://www.iedb.org).
