# Supplementary material for: Comparative Genomics of Field Isolates of Mycobacterium bovis and M. caprae Provides Evidence for Possible Correlates with Bacterial Viability and Virulence
Source: PLoS Negl Trop Dis. 2015 Nov 19;9(11):e0004232. doi: 10.1371/journal.pntd.0004232 (PMC4652870; doi:10.1371/journal.pntd.0004232)
Supplement: S5 Table — (DOCX) [file pntd.0004232.s009.docx]

### **S5 Table.** ESX proteins identified in the mycobacteria genomes.

| **MB1  (Uniprot ID)** | **MB2  (Uniprot ID)** | **MB3  (Uniprot ID)** | **MB4  (Uniprot ID)** |
| --- | --- | --- | --- |
| C6DW30 | C6DW30 | C6DW30 | C6DW30 |
| G0TKS4 | G0TKS4 | G0TKS4 | G0TKS4 |
| H8HUI3 | NF | H8HUI3 | H8HUI3 |
| H8HW13 | H8HW13 | H8HW13 | NF |
| H8I294 | H8I294 | NF | H8I294 |
| L0PV18 | L0PV18 | L0PV18 | NF |
| L0QC69 | L0QC69 | L0QC69 | L0QC69 |
| L7N5C4 | L7N5C4 | L7N5C4 | L7N5C4 |
| M1IRV4 | M1IRV4 | M1IRV4 | M1IRV4 |
| M1J229 | NF | M1J229 | M1J229 |
| O05440 | O05440 | O05440 | O05440 |
| NF | O05449 | O05449 | NF |
| O05459 | NF | NF | O05459 |
| O05460 | O05460 | NF | O05460 |
| O06261 | O06261 | O06261 | O06261 |
| O06267 | O06267 | O06267 | O06267 |
| O06317 | O06317 | O06317 | O06317 |
| O33354 | O33354 | O33354 | O33354 |
| O53687 | O53687 | O53687 | O53687 |
| O53688 | O53688 | O53688 | O53688 |
| O53689 | O53689 | O53689 | O53689 |
| O53689 | O53689 | O53689 | O53689 |
| O53696 | O53696 | O53696 | O53696 |
| O53933 | O53933 | O53933 | O53933 |
| O53944 | O53944 | O53944 | O53944 |
| O53946 | O53946 | O53946 | O53946 |
| O69732 | O69732 | O69732 | O69732 |
| O69733 | O69733 | O69733 | O69733 |
| O69734 | O69734 | O69734 | O69734 |
| O69735 | O69735 | O69735 | O69735 |
| O69736 | O69736 | O69736 | O69736 |
| O69740 | O69740 | O69740 | O69740 |
| O69741 | O69741 | O69741 | O69741 |
| NF | O69742 | NF | NF |
| O69743 | O69743 | O69743 | O69743 |
| O69744 | O69744 | O69744 | O69744 |
| O86362 | O86362 | O86362 | O86362 |
| P0A566 | P0A566 | P0A566 | P0A566 |
| P0A568 | P0A568 | P0A568 | P0A568 |
| P0A570 | P0A570 | P0A570 | P0A570 |
| P63744 | P63744 | P63744 | P63744 |
| P65087 | P65087 | P65087 | P65087 |
| P96212 | P96212 | P96212 | P96212 |
| P96213 | P96213 | P96213 | P96213 |
| Q8VJW6 | Q8VJW6 | Q8VJW6 | Q8VJW6 |
| Q933K8 | Q933K8 | Q933K8 | Q933K8 |
| R4MN35 | R4MN35 | R4MN35 | R4MN35 |
| S5EZJ9 | S5EZJ9 | NF | S5EZJ9 |
| V2V7U1 | NF | V2V7U1 | V2V7U1 |
| V2VSA7 | NF | V2VSA7 | V2VSA7 |

Uniprot accession numbers are shown for each sequence. Abbreviation: NF, not found.
